# National Tuberculosis Coalition of America Guidelines for Respiratory Isolation and Restrictions to Reduce Transmission of Pulmonary Tuberculosis in Community Settings

**DOI:** 10.1093/cid/ciae199

**Published:** 2024-04-18

**Authors:** Maunank Shah, Zoe Dansky, Ruvandhi Nathavitharana, Heidi Behm, Shaka Brown, Lana Dov, Diana Fortune, Nicole Linda Gadon, Katelynne Gardner Toren, Susannah Graves, Connie A Haley, Olivia Kates, Nadya Sabuwala, Donna Wegener, Kathryn Yoo, Joseph Burzynski

**Affiliations:** Division of Infectious Diseases, Department of Medicine, Johns Hopkins University, School of Medicine, Baltimore, Maryland, USA; Division of Infectious Diseases, Department of Medicine, Johns Hopkins University, School of Medicine, Baltimore, Maryland, USA; Division of Infectious Diseases, Beth Israel Deaconess Medical Center and Harvard Medical School, Boston, Massachusetts, USA; TB Program, Oregon Health Authority, Portland, Oregon, USA; We Are TB, Stop TB USA, Atlanta, Georgia, USA; Washington State Department of Health, Shoreline, Washington, USA; National Tuberculosis Coalition of America, National TB Nurse Coalition, Atlanta, Georgia, USA; We Are TB, Stop TB USA, Atlanta, Georgia, USA; Public Health Seattle and King County Tuberculosis Control Program, Seattle, Washington, USA; San Francisco Department of Public Health, San Francisco, California, USA; Division of Infectious Diseases, Department of Medicine, Vanderbilt University Medical Center, Nashville, Tennessee, USA; Division of Infectious Diseases, Department of Medicine, Johns Hopkins University, School of Medicine, Baltimore, Maryland, USA; Berman Institute of Bioethics, Johns Hopkins University, Baltimore, Maryland, USA; Minnesota Department of Health, St. Paul, Minnesota, USA; National TB Coalition of America, Atlanta, Georgia, USA; Texas Department of State Health Services, Tuberculosis and Hansen's Disease Unit (TXDSHS), Society of Epidemiologists in Tuberculosis Control (SETC), Texas, USA; New York City Department of Health and Mental Hygiene, New York, New York, USA

**(See the Invited Commentary by Goswami and Reed on pages e715–7.)**

## Abbreviations

ACET: Advisory Council for the Elimination of Tuberculosis
AFB: acid-fast bacilli
AII: airborne infection isolation
ATS: American Thoracic Society
ATT: anti-tuberculosis therapy
CASS: cough aerosol sampling
CDC: US Centers for Disease Control and Prevention
CFU: colony-forming units
COE: certainty of evidence
COVID-19: coronavirus disease 2019
Ct value: cycle threshold value
DOT: directly observed therapy
DST: drug-susceptibility testing
DTBE: Division of Tuberculosis Elimination
EBA: early bactericidal activity
ESG: Evidence Synthesis Group evidence to recommendation
FMS: face-mask sampling
GDG: Guideline Development Group
HEPA: high-efficiency particulate air
IDSA: Infectious Diseases Society of America
IGRA: interferon-gamma release assay
NAAT: nucleic acid amplification test
NCHHSTP: National Center for HIV, Viral Hepatitis, STD and TB Prevention
NPI: non-pharmaceutical public health intervention
NTCA: National Tuberculosis Coalition of America
PAS: para-amino salicylic acid
PICO: Population, Intervention, Comparison, Outcome
PWTB: person or persons with TB
RCT: randomized controlled trial
RIR: respiratory isolation and restriction
RS: rRNA synthesis
SARS-CoV-2: severe acute respiratory syndrome coronavirus 2
TB: tuberculosis
TST: tuberculin skin test
UN: United Nations
vDOT: video directly observed therapy
WHO: World Health Organization
XDR: extremely drug-resistant

## 1 INTRODUCTION AND SCOPE OF GUIDELINES

The guidelines outlined in the following document are recommendations from the National Tuberculosis (TB) Coalition of America (NTCA; formerly the National TB Controllers Association) on public health practices related to respiratory isolation and restrictions (RIRs) for persons with pulmonary TB (PWTB) in community settings (ie, non-healthcare and non-congregate settings). Healthcare settings, by contrast, include hospitals, nursing homes, and other healthcare facilities and are not covered in this set of guidelines. This guidance also may not reflect considerations for other types of congregate and institutional settings, including homeless shelters and correctional facilities, which have historically been locations at increased risk of transmission of TB. The intent of these guidelines is to fill the gap that stems from a lack of existing US national-level guidance for community settings. The guidelines in this document incorporated ethical and legal principles outlined in the prior “TB Control Laws and Policies’ document endorsed by the NTCA, Advisory Council for the Elimination of Tuberculosis (ACET), and Centers for Disease Control and Prevention (CDC) [1]. The guidelines were informed by a balanced representation of TB survivors and advocates, TB clinicians, TB public health practitioners, TB nurses, TB epidemiologists, researchers, and bioethicists.

These guidelines are intended to be used by individuals within TB public health programs to make decisions related to community-based RIRs for public health purposes and may include, but are not limited to, clinicians, health officers, or other designated practitioners at state or local health departments. Guidance for the prevention of TB in healthcare settings and high-risk congregate living facilities has been provided elsewhere [2, 3]. TB programs are encouraged to update or develop local guidelines and practices and involve physician and public health consultants with TB expertise, to ensure that local practices reflect current scientific evidence and concepts and recommendations outlined in this work.

This document is organized into an Executive Summary (abridged guidelines), as well as the Full Recommendations. The Executive Summary outlines the background, methodology, and key findings and provides a summary of recommendations (Table 1), explanation of community-based RIRs (Table 2), as well as a schematic representation of the guidelines (Table 3), and a quick reference for implementation purposes (Tables 4 and 5). More detailed information about each of these sections is found in the complete Full Recommendations section.

**Table 1.** Recommendations for Community-Based Respiratory Isolation and Restriction for Persons With Tuberculosis

Recommendation 1: Goals of RIR	1.1. The decision to recommend TB RIR should consider the potential benefits and harm for both the community and the PWTB.
Recommendation 2: Defining RIR (Table 2)	2.1. RIR in community settings should be conceptualized as a spectrum of tailored restrictions that are individualized for specific circumstances (Table 2).
Recommendation 3: Determining infectiousness and transmission risk (Figure 1)	3.1. Prior to effective^a^ ATT initiation, PWTB with higher respiratory bacterial burden (ie, sputum smear and/or NAAT positivity, cavitation on chest imaging) may be considered as relatively more infectious than those with lower bacterial burden, with individual variability.
	3.2. PWTB on less than 5 days of effective ATT should be considered relatively more infectious than those on longer durations of effective^a^ therapy.
	3.3. PWTB on effective^a^ ATT for at least 5 days should be considered noninfectious or as having a low likelihood of infectiousness, regardless of sputum bacteriologic status during ongoing ATT (ie, smear microscopy or culture status), with certain exceptions.^b^
	3.4. Overall risk of transmission to others should consider both a PWTB's infectiousness, as well as other factors including the environment of potential exposures, durations of exposure, and biological susceptibility of contacts.
Recommendation 4: Determining RIR (Table 3)	4.1. RIR is not recommended for persons with noninfectious forms of TB (ie, localized extrapulmonary TB without pulmonary involvement, as confirmed by sputum bacteriologic studies and/or chest imaging).
	4.2. People with pulmonary TB on effective^a^ ATT and a low likelihood of infectiousness should not have restrictions in most circumstances (ie, RIR should be removed, if present),^b^ with individual exceptions for situations involving higher-risk community settings and populations (eg, children <5, immunosuppressed individuals).
	4.3. Community-based RIR may be considered for PWTB who have higher infectious potential in which there is judged to be higher risk of transmission to the community.
Recommendation 5: Determining level of RIR (Table 3)	5.1. When community-based RIR is indicated for a PWTB, a moderate or midlevel range of RIR (Table 2) should be considered appropriate in most circumstances, with individual exceptions.
	5.2. Specific RIR levels (eg, low, moderate, or extensive; Table 2) and duration for PWTB should be reassessed routinely (at least weekly) and may be modified based on individual considerations or changing circumstances.
	5.3. When RIR is implemented, support should be provided to patients to mitigate anticipated and experienced harms.

Abbreviations: ATT, anti-tuberculosis therapy; NAAT, nucleic acid amplification test; PWTB, person or persons with tuberculosis; RIR, respiratory isolation and restriction; TB, tuberculosis.

^a^Effective ATT is defined as a recommended multidrug regimen to which the organism is susceptible or anticipated to be susceptible.

^b^No single test or ATT duration universally predicts noninfectiousness. While there is individual variability in infectiousness, available evidence indicates most PWTB are unlikely to transmit to others after the first few days (24–72 h) of ATT initiation. Recognizing pragmatic considerations for time needed to assess ATT adherence and tolerance, and conduct clinical and public health evaluation, community-based RIR can be discontinued in most circumstances after 5 days of ATT, with certain exceptions. Additional factors that may be considered when assessing ongoing infectiousness include the initial bacterial load (eg, high pre-ATT bacterial burden), adequacy of ATT regimens (bactericidal and sterilizing potential; drug susceptibility), and/or adherence and clinical response to ATT; sputum bacteriologic status during ATT is not expected to provide information that reliably correlates with infectiousness. Individualized extensions may be warranted in settings and situations with higher risk or consequence of transmission, including exposures to children <5 years and immunosuppressed or other vulnerable populations. The optimal duration of RIR in such situations is uncertain and should balance community risks and benefits. While PWTB on longer durations of ATT are expected to be less infectious than those on shorter durations, longer durations of RIR are anticipated to result in increased patient harms. Expert consultation or additional review should be sought when RIR has extended beyond 14 days.

**Table 2.** Spectrum of Respiratory Isolation and Restriction for Persons With Tuberculosis in a Community-Based Setting

Extensive restriction	Individuals should strictly limit their movement to an agreed-upon location, such as a home or other residence. Any exceptions to extensive RIR should be discussed and agreed upon with the local health department officials. When an individual leaves the primary site of RIR (such as for a healthcare visit), additional measures to reduce TB transmission risk may be warranted, including but not limited to, personal protective equipment (eg, N95 masks) for close contacts, face masks (ie, surgical masks, KN95, N95) for the PWTB, and efforts for improved ventilation (eg, open windows during transportation in cars, negative-pressure rooms or HEPA filters). Visitors not living in the residence should be avoided unless approved by the local health department and should wear personal protective equipment (eg, N95).
Midlevel/moderate restrictions	Individual spends majority of time at an agreed-upon location, such as a home or residence. Individual may leave the location for most outdoor activities and some indoor activities deemed essential, as determined through discussion with public health department officials: Individual may engage in most activities in outdoor or well-ventilated environments^a^; Strategies to minimize aerosols including wearing a mask (ie, surgical mask, KN95, N95) should be utilized for indoor activities, particularly if there is contact with previously unexposed individuals; Indoor activities should avoid prolonged (eg, multiple hours), or repeated close contact with others, particularly individuals not previously exposed or vulnerable populations (eg, children, immunosuppressed individuals)^a^; Indoor activities in settings of poor ventilation or dense populations should be avoided^a^; In settings at higher risk of transmission (eg, healthcare visit), or potential risk of transmission to vulnerable populations (eg, immunosuppressed, children), additional measures to reduce transmission risk may be warranted, including but not limited to, personal protective equipment (eg, N95 masks) for close contacts, face masks (ie, surgical masks) for the PWTB, and efforts for improved ventilation (eg, negative-pressure rooms or HEPA filtration systems). Visitors should be avoided unless approved by the local health department and should wear personal protective equipment (eg, N95).
No restriction	Individuals have no restrictions and may engage in daily activities as usual, irrespective of setting or potential contacts.

Levels should not be considered absolute but represent a framework for individual judgments. The duration of restrictions should consider both the individual's infectiousness (Figure 1, chart A), as well as the potential risks and consequences of transmission to others (Figure 1, chart B) and are summarized in Table 3.

Abbreviations: HEPA, high-efficiency particulate air; PWTB, person or persons with tuberculosis; RIR, respiratory isolation and restriction; TB, tuberculosis.

^a^Studies suggest that transmission risk is lower in outdoor settings and locations with natural ventilation and/or UV light, compared with shared indoor airspace and closed-ventilation systems. Other factors that influence the likelihood of transmission from a PWTB to an exposed contact include duration, frequency, and proximity of exposures. There is no minimum duration, frequency, or proximity of exposure that defines likelihood of transmission. While short durations or infrequent exposure could lead to *Mycobacterium tuberculosis* infection, many contacts are not infected after longer durations (weeks to months) of intensive exposure. Overall, the probability of transmission is expected to increase with more frequent (ie, daily) contact for longer durations (eg, >8 h), in indoor settings at close proximity.

**Table 3.** Integrated Schematic and Decision Aid to Support Community-Based Respiratory Isolation and Restriction Recommendations for Individuals With Pulmonary Tuberculosis

Recommendation 3: Determining Infectiousness			Recommendation 4: Determining RIR	Recommendation 5: Level of RIR	Notes
ATT status	Pretreatment respiratory bacterial burden^a^	Assessment of individual infectiousness^a,b^	Is RIR indicated?^c^	What level of RIR to choose? (Rec 2; Table 2)	Specific recommendations should balance community and patient risks and benefits (Rec 1)
Pretreatment	High	Highest (Rec 3.1)	Yes (Rec 4.3)	Extensive	Support should be provided to mitigate harm to PWTB (Rec 5.3)
	Low	Moderate (Rec 3.1)	Yes (Rec 4.3)	Extensive or moderate (Rec 5.1)	
Treatment ≤5 d	High	Moderate (Rec 3.2)	Yes (Rec 4.3)	Moderate (Rec 5.1)	
	Low	Moderate/low (Rec 3.2)	Yes (Rec 4.3)	Moderate (Rec 5.1)	
Treatment >5 d	High	Low (Rec 3.3)^b^	Not indicated in most situations (Rec 4.2)^d^	None	Individual exceptions to continue RIR may be considered (Rec 5.2)^d^
	Low	Lowest (Rec 3.3)		None	

Abbreviations: ATT, anti-tuberculosis therapy; NAAT, nucleic acid amplification test; PWTB, person or persons with tuberculosis; Rec, Recommendation (from Table 1); RIR, respiratory isolation and restriction; TB, tuberculosis.

^a^Prior to treatment, assessment of respiratory bacterial burden may include sputum smear microscopy testing (smear positivity and grade), NAAT (lower cycle thresholds may indicate higher bacterial burden), and/or cavitation. Before ATT initiation, higher bacterial burden (and strength of aerosolization) may be associated with greater infectious potential (see Figure 1, chart A, *y*-axis).

^b^There is individual variability in the rate of decline of infectiousness following ATT initiation, but available evidence suggests rapid decline in infectiousness after treatment initiation. Most individuals should be considered to have a low likelihood of infectiousness after 5 days of effective ATT, defined as a multidrug treatment regimen to which the organism is susceptible or anticipated to be susceptible (see Figure 1, chart A, *x*-axis). Factors that may be associated with a longer duration of infectiousness may include high pretreatment respiratory bacterial burden (eg, cavitation, based on initial sputum smear and/or NAAT status), bactericidal and sterilizing activity of the treatment regimen, and adherence and tolerance of treatment. Final decisions on RIR should also include an assessment of net transmission risk to others in the community (see Figure 1, chart B).

^c^The decision to recommend TB RIR should consider the potential benefits and harm for both the community and the PWTB (Rec 1.1).

^d^Additional restrictions or longer duration may be considered in some scenarios of known or suspected drug-resistant TB, higher-risk community settings (eg, longer duration, frequency, and increased proximity of previously unexposed contacts in indoor settings with poor ventilation), potential exposure to vulnerable contacts (eg, children <5, immunosuppressed individuals), slow or inadequate clinical response to ATT, or inadequate adherence to daily ATT. Specific recommendations should balance community well-being and patient impact. Additional review or expert consultation is warranted when RIR is extended beyond 14 days.

**Table 4.** Implementation Aid for Initial Determination of Community-Based Respiratory Isolation and Restriction for Newly Diagnosed Persons With Tuberculosis

Step	Assessment	Notes and Recommendations
Assess infectiousness and transmission risk (see Rec 3)	Review initial chest imaging: presence or absence of cavitation	Individuals without prior imaging or bacteriologic evaluation of TB involvement in the respiratory tract should have assessment that includes a chest radiograph and expectorated sputum evaluation using smear microscopy, NAAT, and culture, when possible. Individuals with pretreatment cavitation or sputum smear or NAAT positivity may have a higher initial bacterial burden and may be relatively more infectious than individuals with sputum smear and/or NAAT-negative samples (see Rec 3.1). Children under 10 y, particularly those with limited bronchial, laryngeal, or pulmonary involvement and minimal cough, are not generally regarded as infectious.
	2. Review initial sputum or respiratory bacteriologic studies	
	3. Review initial DST and treatment regimen	Molecular DST should be used, when possible, to rapidly assess at least rifamycin susceptibility (eg, GeneXpert MTB/RIF [Cepheid Inc, Sunnyvale, California]). If rapid molecular or phenotypic DST is unavailable, initial drug selection and determination of ATT effectiveness is based on the epidemiologic likelihood of drug resistance and may consider clinical response to treatment. Individuals with suspected or identified drug resistance should have additional evaluation (eg, CDC Molecular Detection of Drug Resistance testing; phenotypic DST to first- and second-line drugs) to confirm the effectiveness of a chosen ATT regimen.
	4. Consider risk of transmission to the community (answering yes to 1 or more suggests relatively higher risks of community transmission; see Figure 1, chart B)	(See Rec 3.4; see Figure 1, chart B) Assess housing—Is there shared ventilation with individuals who have not been previously exposed? If so, assess if transmission risks can be mitigated (ie, wear a surgical mask or minimize time spent in shared environment with others), or consider alternative housing options. Assess employment, school setting, social activities, and other settings where PWTB will spend time—Is there likely to be prolonged (eg, multiple hours) or repeated contact in close proximity (eg, same room) with others, particularly previously unexposed?^a^ Is there likely to be contact with vulnerable populations (children, immunosuppressed individuals, such as in healthcare settings)? Are there higher-risk environments (consider ventilation, space, density of occupants) where the PWTB is anticipated to spend time?
2. Determine whether community-based RIR is indicated (see Rec 4)	Does the PWTB have evidence of pulmonary TB? Is the individual infectious and at high risk of transmission in the community? Assess potential harms of RIR for PWTB	RIR is not indicated for individuals with localized extrapulmonary TB in whom TB of the respiratory tract has been excluded (see Rec 4.1). Community-based RIR is indicated for most PWTB with pulmonary or respiratory involvement who have not completed at least 5 days of effective treatment (see Rec 4.3). Table 5 outlines decisions for the duration of community-based RIR. The decision to recommend RIR should consider the potential benefits and harm for both the community and the PWTB (Rec 1.1).
3. Determine level of RIR (see Rec 5)	Individualize RIR based on specific circumstances, balancing community and individual benefits and harms (see Table 2 and Table 3 for a framework of restrictions)	Prior to implementation, community-based RIR should be discussed with the patient to identify potential harms that can be modified.^b^ The least-restrictive measures to achieve goals of reducing community TB transmission should be used based on the characteristics of the setting and infectiousness of the PWTB (see Rec 5.1). Home or community-based RIR is preferred, when possible, over hospital-based RIR. In most instances, outdoor activities that are low risk for TB transmission should be allowed. More extensive restrictions may be warranted prior to treatment initiation, with moderate restrictions once on effective ATT. The intensity and duration of RIR should be determined based on specific individual considerations and clinical and community context.
4. Assess support services (see Rec 5)	Evaluate for negative impacts of community-based RIR (see Supplementary Appendix 1)	Appropriate supportive services should be used to minimize the harm of RIR, such as provision of nutritious, culturally appropriate food, phone or video contact with friends, and remote access to school and employment where possible (see Rec 5.3).

Abbreviations: ATT, anti-tuberculosis therapy; CDC, Centers for Disease Control and Prevention; DST, drug-susceptibility testing; MTB, *Mycobacterium tuberculosis*; NAAT, nucleic acid amplification testing; PWTB, person or persons with tuberculosis; Rec, Recommendation (from Table 1); RIR, respiratory isolation and restrictions; TB, tuberculosis.

^a^Studies suggest transmission risk is lower in outdoor settings and locations with natural ventilation and/or UV light, compared with shared indoor airspace and closed-ventilation systems. Other factors that influence the likelihood of transmission from a PWTB to an exposed contact include duration, frequency, and proximity of exposures. There is no minimum duration, frequency, or proximity of exposure that defines likelihood of transmission. While short durations or infrequent exposure could lead to *Mycobacterium tuberculosis* infection, many contacts are not infected after longer durations (weeks to months) of intensive exposure. Overall, the probability of transmission is expected to increase with more frequent (ie, daily) contact for longer durations (eg, >8 h), in indoor settings at close proximity.

^b^See Supplementary Appendix 1 for potential considerations to ensure multiple dimensions of patient experiences are considered.

**Table 5.** Implementation Aid to Assess Duration of Restrictions for Persons With Tuberculosis for Whom Community-Based Respiratory Isolation and Restriction Has Been Implemented

Step	Assessment	Notes and Recommendations
Assess how long PWTB has been under community-based RIR	Has PWTB been under community-based RIR for more than 5 days?	Decisions should be reassessed at least weekly, as well as with change in assessment of infectiousness, and changing circumstances related to patient and community benefits and harms (see Rec. 5.2). Consider additional expert consultation or review when RIR duration has extended longer than 14 days, while ensuring adequate support for PWTB (see Rec 5.3).
2. Assess PWTB infectiousness	Assess duration of verified (ie, DOT or vDOT) treatment. Was ATT considered effective? Infectiousness is expected to progressively decline with ongoing ATT; alternatively prolonged duration of RIR is expected to result in harm for PWTB	Effective ATT is defined as a multidrug regimen to which the organism is susceptible or anticipated to be susceptible. If full DST is unavailable, decisions may be made based on available information (eg, rifamycin susceptibility) and clinical assessment of probability of drug resistance. Most individuals completing at least 5 days of effective ATT have low infectious potential (see Recs 3.2–3.3), and RIR may be discontinued (see Rec 4.2). While ATT rapidly reduces a PWTB's infectiousness there may be individual variability. Available bacteriologic tests do not reliably predict infectious potential during ATT. In some instances of high initial bacterial burden (eg, pretreatment, sputum AFB smear-positive, cavitation), longer treatment durations (eg, 5–14 d) are expected to further reduce a PWTB's infectious potential (see Figure 1, chart A). Clinicians may use individualized judgment in assessing infectiousness based on pre-ATT bacterial burden (ie, initial sputum AFB smear status and cavitation), clinical response to ATT, drug susceptibility, adherence, and duration of ATT. Available data do not support repeated sputum smear microscopy and NAAT testing solely to assess ongoing infectiousness during ATT. Some clinicians may consider repeat sputum bacteriologic labs to monitor ATT response. However, changes to sputum smear, culture, and NAAT test results on ATT may not correlate with a PWTB's infectious potential.
3. Assess community risk of TB transmission	Is there high risk of community TB transmission?	See Step 1, Assessment 4, Table 4
4. Assess potential patient harms	Is patient experiencing harms related to RIR?	There is a lack of validated tools to reliably measure or capture patient harm resulting from RIR. Consider assessment of stigma, financial security, housing, food security, and mental health. Appropriate supportive services should be used to minimize the harm of RIR, such as provision of nutritious, culturally appropriate food, phone or video contact with friends, and remote access to school and employment where possible (see Supplementary Appendix 1).
5. Determine if RIR should be continued	Is there an ongoing high likelihood of infectiousness and risk of community transmission? Are there vulnerable populations to consider, drug resistance, or other special community circumstances?	RIR should be discontinued for most PWTB who are assessed to have low infectious potential (see Recs 4.1–4.2) (eg, after at least 5 days of effective treatment). RIR may be extended based on comprehensive assessment of the PWTB's infectiousness (see above), community risks and consequences of TB transmission, and individual harms. Some considerations that may warrant extended RIR despite a PWTB's low infectious potential include: Anticipated exposures to vulnerable populations including children <5 years (eg, daycares, schools), and immunosuppressed individuals (eg, healthcare settings); Anticipated return to congregate living facilities (eg, homeless shelters) or densely populated environments with poor ventilation^a^; Known or suspected TB drug resistance where the consequences of transmission should be weighed with the harms of prolonged RIR. Decisions to extend RIR should balance individual harms of prolonged restrictions, with anticipated community benefits. Instances where duration has extended beyond 14 days warrant additional review and expert consultation (see Rec 1).

Abbreviations: AFB, acid-fast bacilli; ATT, anti-tuberculosis therapy; DOT, directly observed therapy; DST, drug-susceptibility testing; NAAT, nucleic acid amplification test; PWTB, person or persons with tuberculosis; Rec, Recommendation (from Table 1); RIR, respiratory isolation and restriction; TB, tuberculosis; VDOT, video directly observed therapy.

^a^Studies suggest that transmission risk is lower in outdoor settings and locations with natural ventilation and/or UV light, compared with shared indoor airspace and closed-ventilation systems. Other factors that influence the likelihood of transmission from a PWTB to an exposed contact include duration, frequency, and proximity of exposures. There is no minimum duration, frequency, or proximity of exposure that defines likelihood of transmission. While short durations or infrequent exposure could lead to *Mycobacterium tuberculosis* infection, many contacts are not infected after longer durations (weeks to months) of intensive exposure. Overall, the probability of transmission is expected to increase with more frequent (ie, daily) contact for longer durations (eg, >8h), in indoor settings at close proximity.

## 2 EXECUTIVE SUMMARY

### Background (Abridged)

*Mycobacterium tuberculosis* is a globally prevalent infection that spreads person-to-person via airborne transmission and warrants concerted treatment and prevention efforts. The plan for “Controlling Tuberculosis in the United States” set forth by the CDC, American Thoracic Society (ATS), and the Infectious Diseases Society of America (IDSA) in 2005 included 4 principles to prevent and control TB, covering topics such as prompt reporting, evaluation of contacts, testing and treatment, and transmission prevention in high-risk settings [4]. Within that document it is noted that institutional infection-prevention measures developed in the 1990s were successful in reducing transmission in healthcare settings [4]. The plan did not articulate specific national recommendations for isolation in community settings as a core infection-control measure, but discussed general guidelines to reduce infectivity, which includes prompt and effective anti-tuberculosis therapy (ATT). More recently, the CDC released the Strategic Plan 2022–2026 from the National Center for HIV, Viral Hepatitis, STD and TB Prevention (NCHHSTP) [5]. This Strategic Plan establishes a goal to reduce morbidity and mortality from TB infections, with specific indicators related to latent TB treatment initiation among diagnosed close contacts (of smear-positive cases), without specific metrics related to respiratory isolation or other public health restrictions [5]. The CDC's Division of Tuberculosis Elimination (DTBE) also released a 2022–2026 Strategic Plan that aligns with the goals of the plan from the NCHHSTP but also does not specifically address community-based RIR practices [6].

Nonetheless, for several decades, respiratory isolation of PWTB residing in the community has been used with a goal of preventing the transmission of TB in the United States, with the passage of state and local laws to grant public health officers the authority to implement restrictions for PWTB. Consequently, in 2009, a *Handbook for Public Health and Legal Practitioners* was prepared by the NTCA and ACET for the CDC, which articulated principles surrounding public health interventions that may encroach on individual rights. Public health authorities must balance the magnitude of public health risk against the rights of individuals and ensure provisions for substantive and procedural due process [1].

The widespread use of prolonged quarantine, isolation, and social-distancing measures to limit transmission of severe acute respiratory syndrome coronavirus 2 (SARS-CoV-2) during the recent coronavirus disease 2019 (COVID-19) pandemic increased awareness of the potential harms these practices can have on individuals [7, 8]. There is increased recognition of the impact on individuals being placed under strict respiratory isolation for prolonged durations, separated from their friends and families and being restricted from attending school, work, or other activities [9, 10]. Preliminary survey data collected by NTCA further demonstrated heterogeneity in the range of approaches to and decisions around home-based isolation across US public health departments [11]. The NTCA leadership decided there was a need for an ethics-informed framework based on current scientific evidence to guide community-based RIR practices for TB. Consequently, in 2022, the NTCA initiated the process of developing evidence-based guidelines for community-based RIRs for persons with TB of the lungs and respiratory tract (referred to broadly as “pulmonary TB” throughout the rest of this document) [12, 13].

### Methodology (Abridged)

A Guideline Development Group (GDG) was created by the NTCA to include representation and expertise from TB programs in the United States and its territories and freely associated states representing high, medium, and low TB-burden jurisdictions, the National Society of TB Clinicians (NSTC), National TB Nurse Coalition (NTNC), Society for Epidemiology of TB Control (SETC), We Are TB (a patient advocacy group), and bioethicists (Johns Hopkins Berman Institute for Bioethics). An Evidence Synthesis Group (ESG) was also created to evaluate scientific literature to inform the GDG. PICO (Population, Intervention, Comparison, Outcome) questions as well as specific additional review questions related to infectiousness of PWTB were established. A systematic review was conducted to summarize the evidence related to the impact of isolation (Intervention) for PWTB (Population) on public health Outcomes (TB incidence and mortality) and on patient Outcomes (mental health, stigma, and costs). The GDG met regularly beginning in late 2022 and through 2023 and engaged in discussions around the history, role, and legal framework for community-based isolation interventions. The GDG reviewed ethical principles of public health decision-making, along with the scientific evidence provided by the ESG, leading to an initial formulation of recommendations. External feedback (ie, outside the GDG and ESG) was invited by the NTCA from nurses, epidemiologists, clinicians, and public health representatives on an initial version of the guidelines, before finalization in February 2024 and endorsement by the NTCA Board and scientific journal submission. The guidelines were endorsed by the Infectious Diseases Society of America in May 2024.

### Key Findings (Abridged)

The ESG's systematic review of the scientific literature found few published studies that evaluated the effect of implementing respiratory isolation in community settings on reductions in population-level TB incidence or mortality. One of the major sources of data on this topic, a clinical trial conducted in the 1950s in Chennai, India, randomly assigned patients with pulmonary TB to initiate ATT either under isolation in a sanatorium or at home. There was no difference in incident TB infection or disease in family contacts [14–19]. Contacts were most likely to develop TB disease within the first 3 months in both arms, suggesting transmission prior to ATT initiation [14]. Although simulation and mathematical modeling studies of isolation interventions suggest that modest reductions in overall population-level TB transmission may be possible, isolation was rarely modeled without other interventions and robust data to guide the assumptions behind the models were often lacking [20–22]. In contrast to limited evidence regarding the impact of isolation on population-level TB outcomes, several published studies suggested that specific isolation practices can have adverse and potentially long-lasting effects on patient mental health and well-being, with a disproportionate impact on marginalized populations [23–31].

It is important to consider, however, that the evaluation of respiratory isolation as a population health intervention to reduce TB transmission is challenging, with limited existing data and current scientific methods. Notably, the absence of evidence is not equivalent to evidence against benefit. A scoping review was therefore conducted to assess current evidence related to the association of sputum smear microscopy results, cough, cavitary disease on chest radiograph, and ATT initiation with potential infectiousness. Overall transmission risk from a PWTB to another individual depends on multiple factors in addition to an individual's infectious potential. Such factors could include setting ventilation, duration and proximity of exposure, and susceptibility of each contact to *M. tuberculosis* infection (Figure 1) [32].

**Figure 1.**
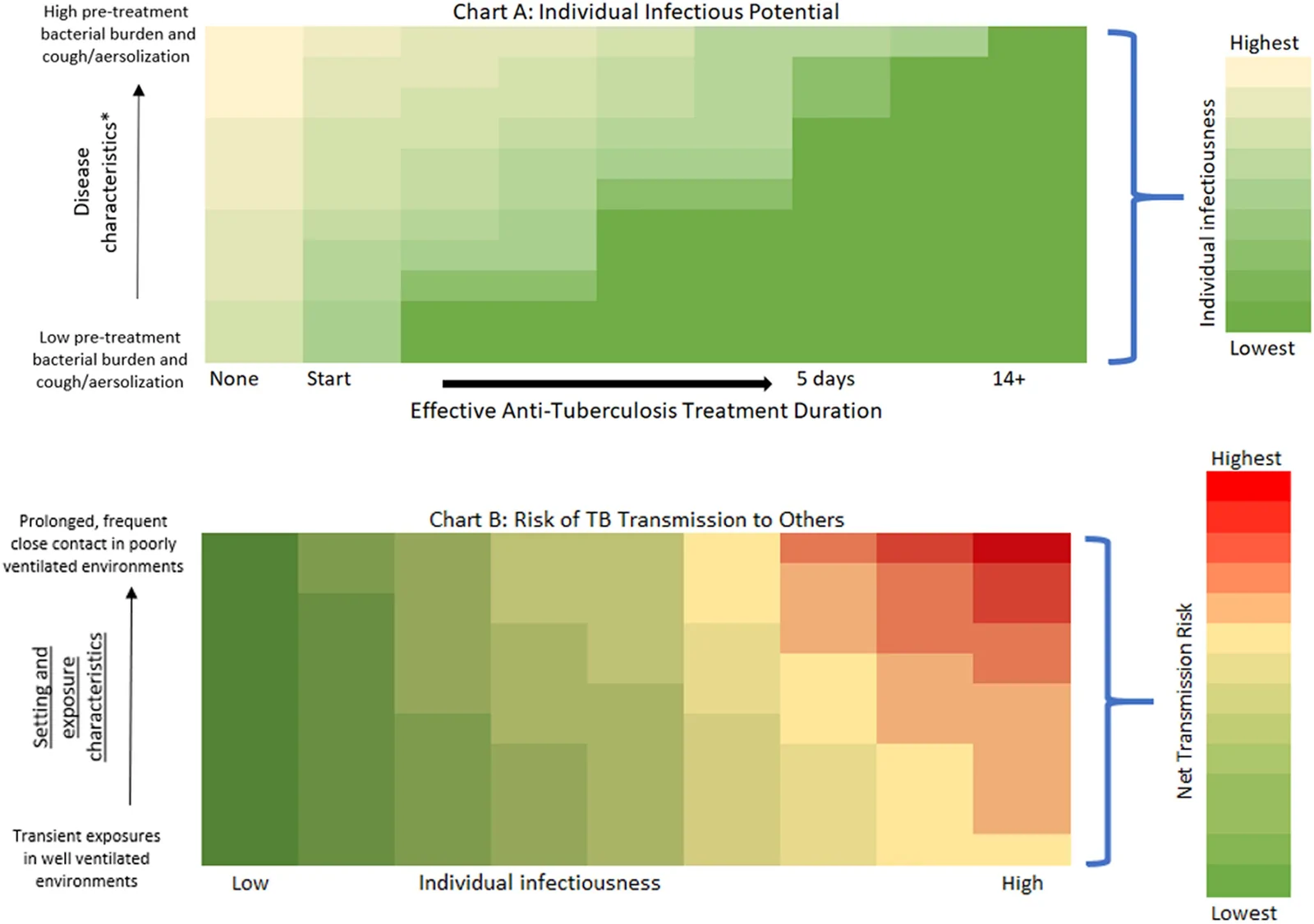
Assessing individual infectious potential and subsequent risk of transmission to others (charts *A* and *B*). Chart A describes the relative infectious potential (ie, degree of infectiousness) of an individual with pulmonary TB. The colors correspond to the relative degree of infectiousness, wherein darker green corresponds to states in which a person is noninfectious or has very low infectious potential, irrespective of sputum bacteriologic status. Prior to anti-tuberculosis treatment initiation, cough aerosol production and measures of bacterial burden (ie, sputum smear microscopy status) may correspond to an individual's infectious potential to others. Once anti-tuberculosis treatment has been initiated, there is rapid decline in infectiousness, irrespective of bacterial burden. The exact duration of anti-tuberculosis treatment that renders a person noninfectious is subject to individual variability. This chart is only an illustrative schematic demonstrating the rapid reduction in infectiousness with effective anti-tuberculosis treatment based on inferences from available bacteriologic studies, human-to-guinea pig transmission studies, and available epidemiologic data. Chart B describes overall risk of transmission based on both an individual's infectious capacity (*x*-axis, use chart A to determine placement along the *x*-axis of chart B) and other factors (*y*-axis), which include the environment in which potential TB exposures occur, duration of exposures, and susceptibility of a contact to infection. Abbreviation: TB, tuberculosis. *There is no single measure that correlates uniformly to bacterial burden or potential aerosol generation that would enable reliable prediction of the infectiousness of a person with TB. Common measures utilized in practice for assessing bacterial burden prior to anti-tuberculosis treatment initiation include presence of cavitation on chest imaging, sputum smear microscopy, and sputum nucleic acid amplification testing.

Prior to ATT initiation, semiquantitative measures of *M. tuberculosis* bacillary burden, such as sputum smear microscopy or nucleic acid amplification test (NAAT) cycle threshold value (Ct value), and clinical (ie, cough) and radiographic measures (ie, cavitation) correlating with the extent of pulmonary disease are associated with higher TB transmission risk [33–39]. Recent studies demonstrate that sputum smear positivity for acid-fast bacilli (AFB) may have poor correlation with other measures of bacillary burden using cough aerosol capture devices and face-mask sampling (FMS); these alternative measures appear to be better predictors of TB transmission to contacts than smear status but are still being evaluated in research studies and not available for clinical use [40, 41].

Studies evaluating the early bactericidal activity (EBA) of combination regimens, including isoniazid, rifampin, and streptomycin, demonstrated dramatic declines in viable *M. tuberculosis* bacilli within a few days of ATT [42, 43]. Cough aerosol sampling (CASS) studies also demonstrate that samples from a high proportion of people producing cough aerosols rapidly become non-culturable within days of ATT initiation [44, 45]. Conversion of sputum AFB smear and mycobacterial culture to negative often occurs after more than 2 weeks of ATT [46]. In vitro viability (ie, ability to detect growth of *M. tuberculosis* in laboratory culture) may not correspond to the ability of the organism to be aerosolized and establish infection in an exposed contact. Experimental data using the human-to-guinea pig transmission model in different settings have consistently indicated that multidrug ATT to which the organism was susceptible reduces infectivity in as little as 48–72 hours, irrespective of sputum AFB smear status [47–51]. The effect of ATT on *M. tuberculosis* likely decreases transmissibility earlier than it inhibits mycobacterial growth in current liquid-based mycobacterial culture systems. Specifically, ATT leads to transcriptomic changes that may affect infectiousness [52–54]. Clinical cohort studies, including those with older ATT regimens that are less effective than current-day ATT, demonstrate a low infection risk for household contacts after 2 weeks of ATT, even if the PWTB is AFB smear positive [55, 56]. Evidence suggests that metrics, such as sputum AFB smear and culture positivity, are not useful predictors of infectiousness after initiation of ATT [57]. There remain limitations in available data to define the exact duration of ATT at which all individuals are predicted to be noninfectious. Several studies have evaluated the effect of ATT on infectiousness at 24–72 hours and others at longer durations such as 2 weeks, but infectiousness at interval time points has not been formally assessed or compared. Nonetheless, the available evidence suggests that infectiousness, and thus transmissibility, decline rapidly and steadily after initiation of effective ATT.

### Recommendations (Abridged Summary)

A series of recommendations were subsequently developed for community-based TB RIR practices based on the available evidence review and a series of discussions regarding the appropriate balance of public health benefits with potential individual harms utilizing an ethics-informed framework. A narrative summary of these recommendations is provided in the text below; specific recommendations are found in Table 1.

### Narrative Summary and Explanation of Recommendations

*Recommendation 1*—*Goals of community-based RIR*: The decision to recommend TB respiratory isolation and restriction (RIR) should consider the potential benefits and harm for both the community and the person or persons with TB (PWTB). Final decisions should be individually tailored, considering relevant patient-specific, setting-specific, and contextual information.
*Recommendation 2—Defining RIR*-*Respiratory isolation restrictions in community settings should be conceptualized as a spectrum of tailored restrictions that are individualized for specific circumstances (from no restrictions to extensive restrictions;* *Table 2*): A framework of categories is proposed: Extensive community-based restrictions for a PWTB include strict limits on movement to a designated location, such as a home, where there is minimal risk of new airborne transmission to previously unexposed persons. Moderate or mid-level restrictions would limit employment, housing, or social/community activities occurring in crowded and/or poorly ventilated indoor spaces, as well as new exposures to vulnerable populations; most outdoor activities where the likelihood of close, prolonged exposure to infectious aerosols is low would be permitted. Low-level or no restrictions would allow most or all activities in community settings.
*Recommendations 3.1–3.4*—*Defining infectiousness and transmission risk (see Table 1 for specific text for 3.1, 3.2, 3.3, 3.4)*: Determining circumstances in which RIR should be considered begins with an assessment of both a PWTB's infectiousness and community transmission risk (Figure 1, charts A and B), recognizing individual and setting variability.

Individuals are most likely to be infectious prior to ATT initiation. Before effective ATT has commenced, PWTB with higher bacterial burden (eg, smear microscopy or NAAT positivity) and greater degree of involvement of airways and the respiratory tract (presence of laryngeal TB or pulmonary cavities) and aerosolization (ie, persistent vigorous cough) are likely to be more infectious compared with those without a cough and low bacterial burden (ie, smear-negative) (Figure 1, *y*-axis; chart A; Recommendation 3.1, Strong Recommendation, Moderate Certainty of Evidence).

Children under 10 years are considered noninfectious in most instances and would not warrant community-based RIR; younger children often lack the ability to generate sufficient infectious aerosols or have lower concentration of organism in respiratory secretions [58]. Children presenting with adult-type TB with cavitation, higher respiratory bacterial burden, and more significant pulmonary or laryngeal involvement should be considered as potentially infectious and treated similarly to adults [58].

There are no tools or laboratory tests that definitively predict the infectiousness of PWTB after initiation of effective ATT. Based on current evidence, effective ATT is suggested to be the primary determinant of infectiousness for the RIR decision-making process. Effective ATT is defined as a multidrug regimen to which the organism is susceptible or anticipated to be susceptible [10, 59]. Rapid molecular drug-susceptibility testing should be conducted, if available, to enable prompt determination of the likelihood of effective ATT. In circumstances when no or only partial drug-susceptibility testing is available, clinical judgment is required, which includes assessing risk factors for drug resistance. Identification or suspicion of rifampin-resistance or other drug resistance may require additional laboratory testing or clinical evaluation to confirm efficacy of a drug-resistant TB treatment regimen before a final determination of infectiousness or RIR.

Once on effective ATT, infectiousness reduces rapidly (ie, 24–72 hours), irrespective of ongoing bacteriologic status of sputum testing, with individual variability [47–50]. The usefulness of microbiological assessment to determine infectiousness during ATT is limited, and infectiousness is expected to decline prior to smear or culture conversion [42, 52, 53]. Individuals not on ATT or on shorter treatment durations (ie, <5 days) are expected to be more infectious compared with those on effective ATT for 5 days or longer (Recommendation 3.2, Strong Recommendation, Moderate Certainty of Evidence).

There is no specific ATT duration that universally predicts that a PWTB is noninfectious. The available evidence from EBA studies, human-to-guinea pig transmission studies, cohort studies, and transcriptomic studies suggests that transmissibility declines rapidly (ie, within 2–3 days) with effective ATT in the majority of PWTB, including those with high pre-ATT bacterial burden assessed by pre-ATT smear positivity or pulmonary cavities on chest X-ray [47–50]. To account for individual variability and time to conduct clinical and public health assessment and to allow rapid molecular drug-susceptibility testing, as well as allow for monitoring of ATT adherence and tolerability, 5 days of continuous effective ATT is recommended as a pragmatic approach to determination of infectiousness.

Consequently, most individuals on at least 5 days of effective ATT should be regarded as having low infectious potential or noninfectious, irrespective of sputum bacteriologic results (including smear microscopy or culture) that are obtained while on ATT (Recommendation 3.3, Conditional Recommendation, Moderate Certainty of Evidence). Longer durations of effective treatment are expected to result in further reductions in infectiousness (Figure 1, chart A, *x*-axis).

Additional factors that may inform determination of infectiousness while on ATT include the initial bacterial load (eg, high pretreatment bacterial burden), adequacy of treatment regimens (bactericidal and sterilizing potential; drug susceptibility), and adherence and clinical response to treatment.

While clinicians commonly utilize sputum bacteriologic testing to inform overall ATT decisions and clinical care for a PWTB (eg, 2-month culture conversion), evidence suggests that bacteriologic studies of sputum (eg, smear microscopy, NAAT, culture) collected while on effective ATT do not reliably predict ongoing infectiousness to inform public health decisions related to community-based RIR.

Transmission is multifactorial, and not solely determined through an assessment of a PWTB's infectiousness. In addition to a PWTB's infectious potential, exposures within indoor settings with poor ventilation, or longer durations of exposure between a PWTB and contacts particularly to vulnerable populations, may constitute a higher risk of transmission to others, compared with outdoor settings and more transient or shorter durations of exposure (Recommendation 3.4). Figure 1 provides a visual schematic assessing both individual-level infectiousness (Figure 1, chart A), and overall risk of transmission to others (Figure 1, chart B), which should be jointly considered in making determinations of community-based RIR. Following an assessment of infectiousness and community transmission risk, public health programs can determine whether community-based RIR is indicated (see Recommendation 4).

*Recommendations 4.1–4.3—Determining if RIR is indicated (see Table 1 for specific text)*-*Scenarios in which there is higher individual infectiousness and/or community risk of transmission may warrant community-based RIR (Table 3 provides an integrated decision-aid and Table 4 and Table 5 provide stepwise implementation outlines)*: RIR is not recommended for individuals with noninfectious forms of TB (ie, extrapulmonary TB in which imaging and sputum bacteriologic studies do not indicate the presence of pulmonary TB) (Recommendation 4.1).

Similarly, restrictions are not recommended (or should be discontinued if in effect) for most individuals with low infectious potential (ie, PWTB on effective ATT for >5 days), with individual exceptions for situations involving higher-risk community settings and populations (Table 3; Recommendation 4.2, Conditional Recommendation, Moderate Certainty of Evidence).

Alternatively, community-based RIR is recommended for most individuals with higher levels of infectiousness (ie, prior to initiation of treatment or prior to at least 5 days of effective ATT; Recommendation 4.3, Conditional Recommendation, Low Certainty of Evidence).

The ultimate duration of RIR should be tailored and consider benefits and harms to the community and the PWTB. In many instances, community-based RIR can be discontinued after 5 days of effective treatment, or when there is a determination that a person has low infectious potential.

Exceptions where the duration of RIR is extended beyond 5 days despite treatment may include, but are not limited to, scenarios of known or suspected drug-resistant TB in which there is ongoing laboratory or clinical evaluation to determine appropriate ATT regimen. Other instances where longer durations of RIR may be warranted include situations with settings that are at higher risk for transmission potential (eg, prolonged, frequent, close contact) in poorly ventilated and/or indoor environments or with contact with vulnerable populations, including but not restricted to, children less than age 5 or immunosuppressed persons who have a higher risk of TB infection and progression to TB disease.

Once a decision to implement community-based RIR has been made, the level of restrictions should be tailored to ensure reductions in community TB transmission risk while limiting potential negative consequences to PWTB (see Recommendation 5). Longer durations of RIR are anticipated to result in greater harm to PWTB, while infectiousness is expected to decline further with longer durations (ie, 5 to 14+ days) of effective ATT. Additional expert consultation or review is warranted when RIR durations extend beyond 14 days.

*Recommendations 5.1–5.3—Determining the appropriate level of RIR*: In most instances where community-based RIR is warranted, a moderate or mid-level set of restrictions is appropriate (Recommendation 5.1), with exceptions based on individual circumstances. Specific RIR levels (eg, low, moderate, or extensive; Table 2) and duration for PWTB should be reassessed routinely (at least weekly) and may be modified based on individual considerations or changing circumstances (Recommendation 5.2). When anticipated or experienced patient harms are substantial, public health programs should ensure that the least restrictions necessary are applied to achieve community goals and ensure that provisions for patient support are used to mitigate harms (Recommendation 5.3).

### Conclusions

In order to eliminate TB in the United States, infection-prevention strategies may include community-based RIR as a component of a comprehensive plan that involves early diagnosis and treatment of PWTB, as well as focused testing and treatment of contacts to PWTB. The NTCA developed these evidence-based, ethics-informed guidelines on the usage of RIR in community settings to fill gaps in existing national-level recommendations. Additionally, they provide a framework for health officers or TB programs to implement community-based RIR with a goal to reduce TB transmission while minimizing individual harms, recognizing that each unique circumstance may require tailored decisions. Implementation of community-based RIR requires multifaceted and patient-centric decision-making that incorporates consideration of each of the stated recommendations (Recommendations 1 through 5). An integrated schematic and decision-aid is provided in Table 3. The approach to a newly diagnosed PWTB therefore begins with an assessment of individual infectiousness and community risks in order to determine the appropriate level of community-based RIR (Table 4). Decisions should be revisited routinely with consideration of changes to infectiousness based on effective ATT duration, assessment of impact of community-based RIR on PWTB, and community risks (Table 5). A snapshot of key principles is summarized in Figure 2 to further support implementation and dissemination of these guidelines.

**Figure 2.**
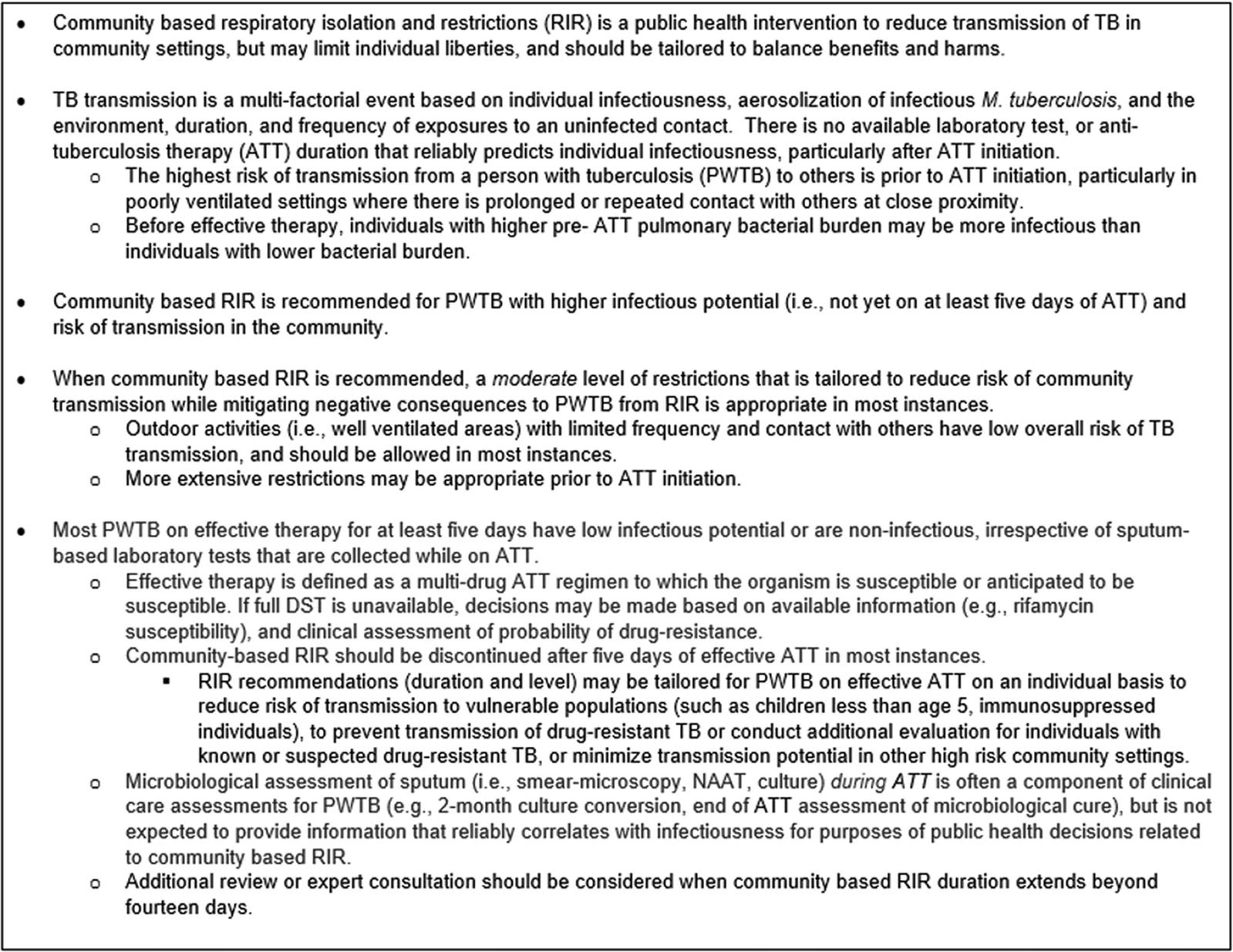
Summary of key principles when considering community-based respiratory isolation and restriction for persons with pulmonary tuberculosis. Abbreviations: ATT, anti-tuberculosis therapy; DST, drug-susceptibility testing; NAAT, nucleic acid amplification test; PWTB, person or persons with tuberculosis; RIR, respiratory isolation and restriction; TB, tuberculosis.

**FULL TEXT OF RECOMMENDATIONS**

## 3 BACKGROUND AND RATIONALE

Tuberculosis is a complex infectious disease with a large impact on public health and individual morbidity and mortality worldwide. In 2021, the United States reported 7882 TB cases, with an incidence rate of 2.4 per 100 000 persons [60]. A total of 600 known TB deaths occurred in the United States for 2020, the highest mortality reported among PWTB nationally since 2006 [60]. Strengthened efforts to decrease TB transmission are urgently needed to mitigate the ongoing impacts of the COVID-19 pandemic that led to an increase in worldwide TB incidence and deaths [61].

The majority of reported TB (71%) in the United States is among non–US-born individuals; however, the incidence rate is increasing for both US- and non–US-born populations [60]. Of TB cases from 2020–2021 with a genotype result, 12% were attributed to recent transmission [60]. An untreated person with active TB is estimated to infect 5–15 people per year through close contact, highlighting the importance of early diagnosis [62, 63]. While TB diagnosis and treatment are considered core transmission-prevention measures, the exact duration of ATT required to render PWTB noninfectious is uncertain [59]. Isolation of PWTB is routinely implemented as a public health strategy to lessen the amount of local transmission and provide community-level benefit. While the impact of TB in the United States is smaller than globally, strong public health response and prevention efforts are still essential to meet TB elimination goals.

Respiratory isolation is an infection-prevention strategy used to reduce transmission of airborne diseases, including TB. When under isolation, a person who is known to have an airborne communicable disease is physically separated from other people to limit disease transmission. This separation typically lasts until an individual is determined to no longer be infectious or has a low likelihood of transmitting *M. tuberculosis* to others [64, 65]. Within US TB programs, the most commonly used duration of respiratory isolation is 14 days after initiation of TB treatment, with some jurisdictions also assessing sputum smear status in de-isolation decisions [11]. This recommendation arose from a 1976 commentary that stated, “people with pulmonary TB are likely no longer infectious after 2 weeks on treatment” based on consensus rather than specific evidence to guide this precise duration [66]. Starting in the 1970s, the US CDC established recommendations for isolation practices in healthcare settings that did not include TB isolation until 1983 [64]. Respiratory isolation remains a widely used TB transmission-prevention measure, despite a limited evidence base when considering community settings [67]. There is growing awareness that diagnosis and management of TB, including the use of respiratory isolation, have a negative impact on PWTB, including stigmatization and mental health concerns [68]. Prolonged durations of isolation and other restrictions aimed at preventing TB transmission may also encroach upon individual liberties of PWTB [30, 31].

The practice of separating PWTB from others dates back to the 1800s. In 1854, the first sanatorium for TB was established in Europe and others were later implemented in the United States [69, 70]. By the early 1950s, there were more than 800 TB sanatoriums in the United States [70]. Notably, these sanitoriums were originally developed to provide a healthier environment for the treatment and healing of PWTB at a time when TB was still considered to be a result of noninfectious etiologies [69]. Specific climates, such as higher altitude, were thought to improve TB-related outcomes for those affected by the disease. Additionally, the sanitoriums provided an environment where people could exercise, prioritize a specific diet, and devote their energy to improving their health [71]. Although unintended, sanatoriums did provide physical separation between PWTB and their families and the public [72]. In the 1960s, the sanatoriums gradually closed as the development of medications and understanding of disease transmission improved [64, 71]. The impact that the sanatoriums had on TB morbidity and mortality is inconclusive [71].

Currently, there are no US national guidelines for implementation of isolation as an intervention to prevent the transmission of TB in community settings. In 2005, the CDC released 2 Morbidity and Mortality Weekly Reports (MMWRs) on recommendations for TB control and elimination: “Controlling Tuberculosis in the United States” and “Guidelines for Preventing the Transmission of Mycobacterium tuberculosis in Health-Care Settings” [2, 4]. The earlier document provides information on criteria for determining individual infectiousness, particularly within healthcare settings. The latter document outlines multifaceted strategies for the prevention of transmission in healthcare settings. For community settings, no specific criteria to initiate or discontinue isolation have been established; these decisions have been based on clinical TB provider or health officer discretion without standardization across jurisdictions. This lack of clear guidance for community-based isolation practices in the United States and internationally has been noted and summarized previously [73].

In the absence of national-level guidance, individual states and local jurisdictions have established their own standards around community-based isolation for PWTB. In many instances, local community-based TB isolation measures mirror those recommended for healthcare facilities [74, 75]. This lack of standardization and consistency creates uncertainty among healthcare providers and TB programs, likely contributing to inequities in the application of isolation restrictions and their effects among PWTB. In addition, this heterogeneous approach may not uniformly consider the tradeoffs between public health benefit and the encroachment of constitutional rights of individuals.

Quantifying and measuring the potential benefits and reductions in TB transmission attributable to isolation practices in the community are difficult. In 2009, a *Handbook for Public Health and Legal Practitioners* was prepared by the NTCA and ACET for the CDC, noting that public health benefits should be balanced against restrictions of constitutional individual rights. The Siracusa Principles, developed by the United Nations (UN) Economic and Social Council in 1984, offer a framework for recommendations that must balance individual harm in the context of public health practice [76, 77]. These principles state that restrictions on human rights under the International Covenant on Civil and Political Rights must meet legal standards, be justified as “strictly necessary,” and use the least-restrictive means to achieve the purpose of the restrictions [76]. Currently, there is no uniform standard between jurisdictions regarding which public health authority or other entity is responsible for making community-based isolation decisions, and processes for patient appeals or legal due process are unclear.

There are limited existing models for the development of public health practice guidelines. While clinical practice guidelines are primarily developed to optimize health outcomes for individuals, public health guidance must consider both individual and population benefits and harms. Both medical and public health perspectives must therefore be weighed when determining decisions related to community-based isolation practices [1]. Strict approaches to TB isolation offer theoretical benefits for limiting transmission among the public in some settings but have not been definitively shown in scientific studies. Alternatively, prolonged isolation may cause harm to the individual, violating basic principles of healthcare delivery [78, 79]. Given the intersectionality between the rationale to isolate a potentially infectious person from others in the community and the potential negative impact on that individual, there are expected tradeoffs that accompany public health–directed restrictions.

The timing and impetus for these guidelines were influenced by several factors. An NTCA survey assessing criteria used for the removal of isolation across US jurisdictions found substantial heterogeneity [11]. Additionally, the 2022 NTCA annual conference included a general session during which survivors of TB recounted their negative experience related to isolation, highlighting potential individual harms of public health restrictions. The timing of this discourse was further influenced by the COVID-19 pandemic, which has drawn intense national scrutiny regarding harms and benefits of non-pharmaceutical public health interventions (NPIs), such as masking, isolation, and vaccination. Inconsistent recommendations related to NPIs issued by local, state, and federal health authorities created confusion among the public and may have limited the uptake and effectiveness of these measures [80]. As a result, there is now increased public awareness and a sharpened focus among healthcare providers regarding the need for clarity, consistency, and justice when considering decisions for community-based RIR for PWTB. In undertaking this work, the NTCA sought to develop evidence-based, patient-centric guidelines for the use of community-based RIR to decrease transmission of *M. tuberculosis*.

## 4 ROLE OF ISOLATION AS A PUBLIC HEALTH INTERVENTION

*Mycobacterium tuberculosis* is transmitted person-to-person through cough-generated airborne infectious aerosols. Patients with only extrapulmonary disease (ie, no concurrent pulmonary TB) are not considered infectious.

Overall goals set forth by CDC's DTBE and by the World Health Organization's (WHO’s) End TB Strategy are to reduce TB incidence by 80% by 2030, reduce TB deaths by 90% by 2030, and to eliminate catastrophic costs to households of PWTB by 2020 [81]. Achieving these goals requires multifaceted interventions to improve diagnosis and management of existing TB and prevent new infection and disease. Modeling suggests that the most effective overall intervention to reduce TB incidence would include a combination of improvements to diagnosis and treat both latent and active TB [82]. Early recognition of TB disease leads to earlier initiation of TB therapy, which, in turn, can reduce potential transmission and avert TB-associated morbidity and mortality.

Within the context of infection-prevention measures, respiratory isolation of PWTB after diagnosis is most studied in healthcare and institutional settings [2, 67]. Facility-based infection-control measures developed in the 1990s were successful in reducing TB transmission in healthcare settings [4].

While most states and local jurisdictions have implemented laws, health codes, or local policy advocating respiratory isolation until a PWTB is deemed noninfectious, there are no overarching national guidelines from the CDC, other health agencies, or professional medical societies. Consequently, local practice and application of respiratory isolation for PWTB are heterogenous in terms of indications, scope, duration, and provisions for an individual's due process [11].

The guidance “Controlling Tuberculosis in the United States” issued by the CDC, ATS, and IDSA in 2005 established 4 core principles: (1) promptly detect and report persons with TB, (2) protect close contacts through contact evaluation and treatment for TB infection, (3) prevent TB through targeted testing and treatment of TB infection, and (4) identify high-risk settings for TB transmission and apply effective infection-prevention measures [4].

Although the 2005 guidance did not outline specific recommendations for the use of TB RIR in community settings, it did outline general measures to reduce a PWTB's infectivity, which includes effective ATT [4]. Specifically, it stated that the majority of patients “who have received treatment for as few as 2 days with the standard regimen (ie, isoniazid, rifampin, ethambutol, and pyrazinamide) could be assumed to have an infective potential that averages 10% of that at the time of diagnosis. After 14–21 days of treatment, infectiousness averages <1% of the pretreatment level” [4].

Furthermore, stringent criteria for the release of a PWTB from respiratory isolation in hospital settings were established: “While in hospital for any reason, patients with pulmonary TB should remain in airborne infection isolation [AII] until they 1) are receiving standard multidrug anti-TB therapy; 2) have demonstrated clinical improvement; and 3) have had three consecutive acid-fast bacilli (AFB)–negative smear results of sputum specimens collected 8–24 hours apart, with at least one being an early morning specimen.” The document does not specify the level of infectiousness (relative to time of diagnosis) at which individuals should remain in isolation in community settings.

The CDC issued a second guidance in 2005, “Guidelines for Preventing the Transmission of *Mycobacterium tuberculosis* in Health-Care Settings,” which reiterated that PWTB should be placed in AII rooms until “the patient has three consecutive, negative AFB sputum smear results” [2]. Many current local and state programs subsequently adopted and extrapolated the same criteria for the duration of respiratory isolation in community settings.

In 2009, a *Handbook for Public Health and Legal Practitioners* was prepared by the NTCA and ACET for the CDC, which articulated principles surrounding public health interventions that may encroach on individual rights [1]. This handbook suggested that public health authorities must balance the magnitude of public health risk against the rights of individuals or groups and ensure provisions for substantive and procedural due process. Acknowledging that legal issues may arise from coercive powers to control persons with TB, the handbook further notes, “Where government action minimally intrudes on non-fundamental individual rights, courts have typically upheld the action so long as there is some ‘rational basis’ for it” [1]. Additionally, it states that “When public health authorities seek to use powers that restrict individual freedoms, fundamental rights of due process, travel, or association may be implicated. In such cases, courts may seek to determine whether (1) government's action advances a compelling state interest that is narrowly tailored, and (2) there are other less restrictive alternatives available to protect the public's health and the individual.” Accordingly, highly restrictive and prolonged RIR would likely be considered more than a minimal intrusion on fundamental individual rights.

Internationally, guidelines also vary with respect to community-based respiratory isolation [10, 73]. The European Center for Disease Prevention and Control's 2017 update to the European Union Standards for Tuberculosis Care recommends respiratory isolation within a hospital setting until sputum evaluation is AFB-smear negative but does not specifically discuss community-based isolation practices [32]. The WHO Regional Office for Europe put forth a consensus document and “guiding principles” on reducing TB transmission in 2018 and 2019 as well, which included a review of the literature related to TB infectiousness [59, 83]. This document suggests that the core question that should inform isolation policies centers on how long it takes for treatment to render a PWTB noninfectious. Their summation of the evidence states that effective treatment “essentially stops transmission” but does not specify a specific duration of treatment for isolation or de-isolation decisions [59]. The 2019 WHO Guidelines on Tuberculosis Infection Prevention and Control does recommend isolation for PWTB, with a focus on healthcare settings; however, no specific duration of isolation is provided and de-isolation should be determined by likelihood of infectivity [78].

In 2019, New Zealand's Ministry of Health released TB guidance that acknowledges the complexity of determining de-isolation practices. They note that “treatment will sufficiently damage mycobacteria to affect transmissibility of organisms much earlier than the damage required to prevent growth in liquid culture.” Consequently, they suggest that a “sensible” approach is for PWTB to remain in isolation for 2 weeks (until laboratory results are complete), “by which time infectivity of even heavily smear-positive patients will have fallen to negligible levels.” This approach is based solely on duration of treatment, without consideration of ongoing sputum bacteriologic status. In summary, they recommend, “Patients with smear-positive pulmonary TB may be removed from isolation after they have had a minimum of two weeks of effective chemotherapy.” Notably, they include factors for earlier removal of isolation, which include absence of cough, low smear score on sputum, and compliance with requests to wear masks outside of isolation [location] [84].

In 2022, the Canadian Tuberculosis Standards included a review of scientific evidence on TB infectiousness and provided updated recommendations for de-isolation [85]. Their review concluded that “the preponderance of data suggests that appropriate treatment rapidly renders PWTB non-infectious, perhaps within a few days of treatment, even for initially smear positive cases,” and “studies also suggest that sputum smear and culture are less predictive of infectiousness once patients are established on effective therapy” [85]. Addressing common TB isolation practices in other settings, the Canadian standards conclude, “The insistence on smear-conversion before lifting airborne precautions may unnecessarily prolong isolation and cause patient harm with little public health benefit” [85]. Nevertheless, this guidance recommends a “minimum of 2 weeks of effective therapy has been completed and there are 3 consecutive negative AFB sputum smears,” but makes provisions for airborne precautions to be discontinued “if there is improvement after completing a minimum of 4 weeks of effective therapy, even if the sputum smears are persistently positive.”

In 2022, the Communicable Diseases Network of Australia set forth guidance that discussed both isolation precautions in the hospital setting and at home. For hospital settings, Australian guidance recommends that PWTB remain in isolation until all of several criteria are met, including reduction in cough, reduced smear burden (or smear negativity), adherence to treatment, and a discharge plan is in place [86]. For home-based isolation, Australian guidance suggested that PWTB should remain isolated “until assessed as being at minimal risk of transmitting infection in consultation with local services” [86].

There is uncertainty regarding the overall proportion of transmission that is avertable by respiratory isolation for PWTB after diagnosis and treatment. Data from modeling suggest that the majority of TB transmission may occur from undiagnosed individuals, a group that would not be addressed by respiratory isolation policies [87]. Data from the CDC's Contact Investigations Report (ARPE Data) show the results of contact investigations from smear-positive cases from 2016–2020 [88]. These data provide some information about the relative infectiousness of TB. Of 38 886 contacts identified in 2019, 5586 contacts were identified as having latent TB infection (∼14%) [88]. The available data are not stratified by duration of exposure, timing of transmission, and contact relative to treatment initiation, or the settings in which exposures may have occurred, limiting inferences. However, these data provide general insights into the relative infectiousness between PWTB and their closest contacts (and often include household contacts with longer durations of exposure), as identified by contact investigation efforts [88].

Ultimately, the population-level goal of isolation for a person with infectious forms of TB is to mitigate avertable transmission and thereby reduce TB incidence, and potential future morbidity and mortality. Estimating the community-level benefit of isolating PWTB is challenging and is influenced by a number of factors beyond an individual's infectiousness. These may include the setting and environment of potential exposures between contacts and PWTB, the duration and frequency of contact, as well as the ability to conduct other public health interventions such as contact investigations and availability of preventative therapy for individuals with recent TB infection. Ultimately, transmission of TB from a PWTB to another individual is therefore a probabilistic event. Consequently, decisions to isolate or separate PWTB from others are not anticipated to have uniform population benefits in all scenarios.

## 5 OBJECTIVES OF NTCA GUIDELINES

The objective of these guidelines is to set forth a framework for public health decision-making regarding community-based RIR to reduce person-to-person TB transmission. These guidelines incorporate current evidence for determining the potential infectiousness of PWTB, effectiveness of respiratory isolation to reduce community-level TB incidence, and assessment of whether individual patient outcomes are affected by the use of RIR. Through development and implementation of an ethics-informed process, these guidelines include recommendations that aim to provide population benefit while maintaining consideration for the individual patient.

Implementation of these recommendations may require evaluation and revision of local or state policies, procedures, laws, and health codes pertaining to TB RIR. It is not feasible to specifically address all diverse patient and community scenarios that may be encountered across all public health jurisdictions. However, these guidelines highlight the importance of an individualized, patient-centric approach within a scientifically based, public health intervention. The guidelines acknowledge that considerations for isolation and public health restrictions may differ based on the availability and feasibility of other elements included in TB-elimination strategies as well as the structure of public health department TB programs—availability of treatment, availability of diagnostic testing, implementation of contact evaluation, and provisions for preventive therapy.

## 6 GUIDELINE DEVELOPMENT METHODOLOGY

The guideline development process was adapted from GRADE (Grading of Recommendations Assessment, Development and Evaluation) methodology and established practices for evidence-based recommendation development, recognizing limitations in applying the GRADE approach to public health interventions (Supplementary Appendix 2) [12, 89]. A GDG was created by the NTCA to include representation from US TB programs from high-, medium-, and low-burden jurisdictions; the National Society of TB Clinicians (NSTC); National TB Nurse Coalition (NTNC); Society for Epidemiology of TB Control (SETC); We Are TB (a patient advocacy group); and bioethicists (Johns Hopkins Berman Institute for Bioethics). This multidisciplinary group allowed for the incorporation of diverse perspectives and TB experiences beyond a provider or health authority's lens. Each representative brought a different clinical, public health, or personal perspective to the group. The GDG members included patients, advocates, clinicians, nurses, epidemiologists, TB researchers, public health program leaders, and policymakers. The GDG met regularly beginning in late 2022 and through 2023 and engaged in discussions around the history, role, and legal framework for community-based isolation interventions, review and consideration of ethical principles of public health decision-making, and consideration of the scientific evidence.

The GDG identified PICO (Population, Intervention, Comparison, Outcome) questions that corresponded to the usage of community-based respiratory isolation for PWTB. The group additionally identified scientific questions related to the determination of infectiousness of persons with tuberculosis, considered essential to guideline development. An ESG was developed separately from the GDG. The ESG conducted a comprehensive literature review to summarize relevant scientific, ethical, and public health literature for the GDG to use as the basis of its recommendations. Strengths of recommendation and certainty of evidence (COE) determinations utilized evidence to recommendation (ETR) frameworks (Supplementary Appendix 2). To ensure objective consideration of the data, the ESG members were available for data presentation and discussion but were not voting members of the GDG.

Public health recommendations impact both patients and communities. For this guidance, individual health outcomes of interest that were considered included TB morbidity and mortality, mental health and stigma, and individual costs (ie, housing, job, and food security). Population-level outcomes included TB (disease and infection) incidence, mortality, and population-level costs.

The specific PICO questions evaluated the impact of respiratory isolation on the following important patient and public health outcomes:

1. Does respiratory isolation improve or worsen outcomes among persons with pulmonary TB when compared with no isolation?
  - Outcomes:
    - TB infection among contacts
    - TB disease among contacts
    - TB mortality
    - TB hospitalization/duration
    - Patient and health system costs
    - Mental health/stigma
2. Does respiratory isolation improve or worsen outcomes among persons with pulmonary TB when compared with masking?
  - Outcomes:
    - TB infection among contacts
    - TB disease among contacts
    - TB mortality
    - TB hospitalization/duration
    - Patient and health system costs
    - Mental health/stigma

In addition to the PICO questions, a scientific review was conducted to evaluate the evidence related to defining the state of infectiousness of a PWTB, related to 4 commonly utilized clinical parameters: sputum smear microscopy, cough, cavitation on chest imaging, and the receipt of effective ATT. Studies involving TB transmission in healthcare settings were excluded, and were recently reviewed as part of a series of reviews commissioned by the WHO to inform prior TB infection-prevention and -control guidelines [67].

The evidence synthesis was completed in mid-2023 and presented to the GDG, which developed an initial set of recommendations for community-based RIR. Details of the COE assessment and strength of recommendation are found in Supplementary Appendix 2. External feedback and review (ie, outside the GDG and ESG) were invited by the NTCA from nurses, epidemiologists, clinicians, and public health representatives on an initial version of the guidelines. Comments and feedback were considered by the GDG and incorporated into a final set of guidelines in February 2024. The guidelines were presented and endorsed by the NTCA Board on 2 February 2024, with subsequent submission for scientific journal publication. The guidelines were endorsed by the Infectious Diseases Society of America in May 2024.

## 7 ETHICAL FRAMEWORK FOR CONSIDERING RESTRICTIONS OR ISOLATION FOR PERSONS WITH TUBERCULOSIS

Public health policy calling for community-based isolation of individuals with potentially transmissible diseases is ethically complex, as it often entails burdens or sacrifices on the part of some individuals to protect the health of the public. The current reality of TB isolation in practice varies across local jurisdictions, often even within a single state. The legal basis for TB isolation arises from powers conferred to state and local authorities, including public health officers, to act to preserve public health, which, in most jurisdictions, generally includes “the isolation of individuals confirmed to have a communicable disease for the period of communicability—to prevent further disease transmission” [1]. These powers must be balanced with constitutional protections including rights of travel and free association as well as a right to due process when individual liberties are infringed. The legal basis for TB respiratory isolation is well summarized in the *Tuberculosis Control Laws and Policies* document, but the potential for tension between individual rights or interests and public health is a central ethical consideration as well [1].

The guideline development process drew on prominent examples of principles for public health ethics to delineate a set of principles and related considerations relevant to TB isolation policy [90–94]. Through a series of collaborative meetings, these principles were reviewed and refined to capture the ethical complexity of this issue and to reflect the diverse perspectives of members of the GDG. The early articulation of ethical principles and values meant that these principles could inform questions for evidence review, provide a lens for interpreting available data, and support the creation of ethically informed guidelines while adhering to a high standard for procedural legitimacy throughout the policy development process.

At the start of the guideline development process, the GDG engaged in a series of discussions to explore core values and goals related to TB RIR. The following rationale for creating a new public health guidance for community-based TB RIR was used as a benchmark and guide throughout the process:

“A community-based TB isolation policy is needed because TB is an airborne infection capable of spreading through communities, and an isolation policy has the potential to reduce or stop the spread of TB. However, current practices may prioritize public health safety to an extent that is detrimental to individual well-being, liberty and social justice. Current practices may be inconsistent and varied, which is detrimental to social justice and procedural legitimacy; and current practices may not reflect all available scientific data.”

The following principles represent a pluralistic system of values considered by the GDG with no strict hierarchy among them:

1. Well-being of individuals and communities or populations: Several dimensions of well-being were considered relevant to respiratory isolation measures, including the health and safety of the individual and the community as well as the livelihood and employment of individuals, as well as social relationships and social cohesion that could be impacted by isolation recommendations.
2. Justice: It was important to consider the fair treatment of individuals and groups of PWTB and the communities in which they live, seeking to recognize differences in perspectives, experiences, and needs of people and communities affected by TB isolation policies, and to lessen rather than exacerbate inequities.
3. Liberty: There was a goal to ensure respect for the freedom of individuals to order their lives as they desire, without infringing upon the liberties of others.

It was anticipated that community-based respiratory isolation measures may lead to conflicts or tensions between values. The GDG identified a set of justificatory conditions for evaluating compromises to 1 value in favor of another. These conditions include the following:

1. Proportionality: Any compromise to 1 value should be justified by at least proportional promotion of another value. A compromise that does not achieve at least proportional promotion of another value would not be justified.
2. Necessity: Any compromise to 1 value should be justified by the necessity for that compromise to achieve the promotion of another value. Any compromise that is not necessary would not be justified.
3. Least infringement: Any compromise to 1 value should be justified by minimizing the infringement of that compromise on the affected value. Any compromise that entails extreme and extraneous infringement on any value would not be justified.

Anticipating limitations in available data and uncertainty in the policymaking process, a set of procedures for navigating uncertainty were proactively considered. These were as follows:

1. Prioritizing information associated with greater certainty, giving greater weight to information derived from a larger body of evidence or higher quality of evidence.
2. Applying the practical wisdom of topic-area experts, with expertise developed through training and education, professional experience, and lived experience.

Finally, recognizing that the development of ethically informed guidelines would necessitate incorporation of ethics into all aspects of guideline development, the NTCA set out to adhere to a high standard for procedural legitimacy, including the following:

1. Representation of stakeholder groups in the policymaking process
2. Transparency regarding all aspects of the policymaking process
3. Management of conflicts of interest and bias
4. Humility and respectful exchange of ideas
5. Clarity of purpose and alignment with purpose

This 3-part framework of ethical principles, justificatory conditions, and approaches to uncertainty, as well as the overarching standards for procedural legitimacy, was developed from the input of the GDG and iteratively refined through cycles of feedback and revision until all members of the group expressed their approval.

## 8 EVIDENCE SUMMARY

The full systematic and literature reviews will be published separately. Key findings that informed the guideline development process are summarized below.

### Systematic Review Methodology

The ESG conducted a systematic literature review to determine the effect of TB respiratory isolation measures on patient and public health outcomes based on established PICO questions. The intervention(s) of interest were any form of respiratory isolation of PWTB to reduce household and/or community transmission. The ESG searched PubMed, EMBASE, CINAHL, Web of Science, Cochrane Central, and WHO–Global Index Medicus using terms for TB and respiratory isolation and also reviewed citations and contacted experts. Study inclusion criteria included studies with data on the effects of respiratory isolation versus no isolation (PICO 1) or masking (PICO 2). Studies that focused on respiratory isolation interventions to reduce facility-based TB transmission were excluded. Study designs could include qualitative, quantitative, or mixed methods and there were no restrictions based on country setting, publication date, or language. Studies were stratified by the following PICO question outcomes: TB infection or disease in contacts, mortality, hospitalization, patient and health system costs, and patient impacts including mental health and stigma. A quality assessment was undertaken using the Mixed Methods Assessment Tool (MMAT). Given the heterogeneity of study designs and outcome measurements, a meta-analysis was not possible; the ESG implemented a convergent mixed-methods approach to integrate quantitative and qualitative findings and assess limitations. Details of overall COE and ETR assessments are found in Supplementary Appendix 2.

### Systematic Review Findings

Overall, a total of 3691 studies were identified in the search, of which 17 were included for analysis. Notably, respiratory isolation was rarely implemented as the sole intervention, and only 1 study had a direct comparison of isolation versus no isolation [14–19]. Given the lack of data to inform the PICO questions, data were included from studies that evaluated the effect of respiratory isolation even if there was no comparator. Of the 17 included studies, there was 1 randomized controlled trial (RCT) [14–19], 1 quasi-experimental pre/post study [95], 1 cohort study [23], 3 modeling studies [20–22], 3 quantitative descriptive studies [96–98], 1 mixed-methods study [25], and 7 qualitative studies [24, 26–31].

### Summary of Benefits and Harms of Community-Based Respiratory Isolation and Restrictions

There was limited direct evidence to quantify the effects of respiratory isolation on community TB transmission (ie, population outcome-incident TB infection). An RCT conducted in Chennai, India (often referred to as the “Madras trial”), assigned patients with pulmonary TB to treatment with a 12-month regimen of daily isoniazid and para-aminosalicylic acid (PAS) that was either initiated in the context of sanatorium isolation or at home without other mitigation measures. The study reported no difference in the risk of incident infection or TB disease among family contacts of patients who initiated TB treatment at home (ie, ongoing exposure) compared with those who initiated treatment under healthcare facility–based respiratory isolation. There was also no difference in mortality among index patients or close contacts between the sanatorium versus home treatment arms [14–19]. Notably, a large proportion of PWTB in both arms had moderate or extensive cavitary disease (78% home, 70% sanatorium) and many had heavy bacillary burden on smear (54% home, 37% sanatorium); drug resistance was rare. The majority of contacts who developed TB disease (17/26; 65%) did so in the first 3 months, suggesting that most of the transmission happens prior to diagnosis.

Three modeling studies offered some indirect evidence to assess the population-level impact of respiratory isolation interventions, each of which suggested that isolation interventions (combined with other transmission-prevention measures) may reduce transmission of TB. However, many assumptions were made during the modeling, including the duration of infectiousness [20–22].

When considering other outcomes, including hospitalization duration, the available evidence suggested that respiratory isolation requirements, often within hospitals (in the context of preventing onward community transmission and typically based on smear conversion), increased hospitalization duration anywhere from 2 weeks to months [96, 98].

The limited available data also suggest that there are high health system costs associated with implementation of respiratory isolation interventions, particularly when resulting in prolonged hospitalization [97]. Specific patient-related costs from hospital isolation include loss of income, food insecurity, increased transportation costs, housing insecurity, and negative impacts on educational progress [26, 27, 30, 98].

Several qualitative studies identified psychological impacts and stigma as a result of respiratory isolation. These negative impacts were compounded in populations with intersecting health disparities, including migrants/immigrants, indigenous people, and incarcerated people [24, 26–31]. Length of hospital stay was used as a surrogate for isolation in some studies. Prolonged hospital stay was associated with poor psychological well-being and the risk of developing depression [23, 25, 98].

Data to inform PICO question 2 were more limited. No studies directly addressed the comparison of masking alone versus respiratory isolation to prevent TB isolation. Preliminary data from 1 prospective quasi-experimental controlled trial evaluated the implementation of a bundle of respiratory-infection measures (separation of the index patient within the home and use of N95 face masks for both index patients and close contacts) and found that this intervention did not significantly reduce TB infection in contacts compared with the standard of care [95]. One modeling study suggested that the use of surgical masks by patients with extremely drug-resistant (XDR) TB could lead to small reductions in overall community XDR-TB transmission. The estimated effect size was larger if masking was combined with reduced hospitalization and more outpatient treatment [22].

A recent systematic review commissioned by the WHO provided data on the role of respiratory isolation in healthcare settings, with a target population of healthcare workers and non–healthcare workers in healthcare settings [67]. The review also considered triage and effective treatment as infection-prevention interventions, with outcomes that included latent TB infection and TB incidence in healthcare settings. Twenty-four studies were considered, of which all but 1 study examined respiratory isolation in combination with other interventions. The effects were variable, ranging from a 1% increase to a 20.5% decrease in latent TB infection. Overall conclusions were that the evidence for the effectiveness of triage, isolation, or effective treatment, alone or in combination, was indirect and low quality [67].

The ESG identified several limitations and knowledge gaps during the guideline evidence review process. The majority of included studies were overall of low to moderate quality, with limited direct evidence to assess the impact of community-based respiratory isolation on the stated patient and population outcomes of interest. There was consequently limited evidence to derive conclusions on the impact of community-based respiratory isolation to reduce TB incidence, mortality, or transmission. Deriving further inferences from the available modeling studies was challenging due to limitations in defining components of the evaluated interventions and other assumptions in model structure or parameter estimates. In contrast, a range of studies were identified that suggest that respiratory isolation may have a negative and potentially long-lasting impact on individual patient mental health and other measures of well-being. Ethical considerations were highlighted by several study authors and included potential human rights violations, specifically on freedom of movement, right to education, justice and equity for marginalized populations, and the need for government/health system support to mitigate negative impacts of prolonged respiratory isolation. Improved study design and reporting to evaluate the effect of interventions on TB transmission should be prioritized, along with expedited development of more accurate tools to measure infectiousness.

### Literature Review of Factors Associated With Infectiousness and Transmission Risk From a Person With Tuberculosis to Exposed Contacts

#### Microbiological and Laboratory Tests

*Sputum AFB smear microscopy*: Positive sputum AFB smear microscopy may be associated with TB transmission risk prior to treatment initiation. Despite its suboptimal diagnostic accuracy, sputum smear microscopy is a recognized measure of *M. tuberculosis* bacillary burden that is used clinically for monitoring treatment response. A positive sputum smear requires the presence of 5000–10 000 AFB/mL, in contrast to culture, which requires 10–100 AFB/mL [. Cohort studies have shown that sputum smear status can be used to predict transmission risk prior to treatment initiation. One prospective cohort study in the United Kingdom demonstrated TB infection in contacts under 14 years old (based on tuberculin skin test TST 99, 100] reactivity) was 65% for contacts exposed to a smear-positive index patient, 27% for smear-negative, culture-positive index patient, and 18% when index patients were smear and culture negative [33]. Similarly, adult and child contacts were more likely to develop TB disease when exposed to a smear-positive index patient (11% and 14%) compared with smear-negative, culture-positive patients (0.07% and 3%) [33]. Another retrospective cohort study from the Netherlands found that TST conversion occurred in 6% of household contacts of smear-negative PWTB compared with 50% of household contacts of smear-positive PWTB [34]. Molecular fingerprinting studies demonstrate that PWTB with sputum smear negativity only account for between 13% and 17% of TB transmission, with a relative transmission rate of 0.19–0.24, compared with those who are smear positive [101, 102]. More recent genomic epidemiology analyses suggest the proportion of transmission attributable to persons with negative sputum smear microscopy may be even lower (8%) [103].
*Nucleic acid amplification tests*: Molecular NAATs are commercially available and can provide qualitative and semi-quantitative results. Semi-quantitative and quantitative results from NAATs may correlate with bacillary burden and predict infectiousness. One commercial assay, Xpert MTB/RIF (GeneXpert; Cepheid, Inc, Sunnyvale, CA, USA), provides semi-quantitative results in the form of cycle thresholds (Ct) at which *M. tuberculosis* DNA becomes detectable. Lower Ct values imply a greater bacterial or nucleic acid burden in the sample. Xpert Ct values are strongly associated with smear status (area under the curve of 0.85; 95% CI, .82–.87] [104, 105]. A unit increase in the Xpert Ct was associated with increased time to culture positivity at weeks 2 and 4 (another surrogate marker for initial bacterial burden) and with lower culture colony grade [105]. Xpert Ct may prove to be a valuable predictor of infectiousness. A multi-center Brazilian cohort study evaluated the association between Xpert MTB/RIF Ct values and *M. tuberculosis* transmission in close contacts of persons with pulmonary TB (80% of whom had a positive smear). The study found that lower baseline Xpert MTB/RIF Ct values were independently associated with a higher likelihood of Quantiferon-Plus (Qiagen, Venlo, the Netherlands) conversion in close contacts (odds ratio [OR], 1.61; 95% CI, 1.12–2.32) [35].
*Cough aerosols*: Positive cultures in cough aerosol appear to be a better predictor of infectiousness than sputum smear microscopy. Cough aerosol sampling systems (CASS), which measure culturable TB bacilli in cough aerosols, may be a better means of predicting infectiousness than sputum smear microscopy [40]. Culture-positive cough aerosols are associated with stronger and more frequent coughing [45, 106–108]. Household contact studies show greater transmission to contacts, measured by higher rates of progression to TB disease and TST or interferon-gamma release assay (IGRA) conversion, from patients with higher numbers of *M. tuberculosis* colony-forming units (CFU) in their cough aerosols and a more severe cough [38, 40, 106]. There is high variability between cough aerosol culture positivity between individuals and thus also likely to be variability in the amount of transmission mediated by cough [40, 107, 108].
*Exhaled* M. tuberculosis *from face-mask sampling (FMS)*: Culturable *M. tuberculosi*s from FMS can predict contact infection. Face-mask sampling is an alternative method of quantifying *M. tuberculosis* bacilli exhaled by people with pulmonary TB [41, 109]. A study conducted in The Gambia showed that 91% of patients with smear-positive pulmonary TB had detectable *M. tuberculosis* in face-mask samples with high variation in IS6110 copy numbers; a high face-mask *M. tuberculosis* level (≥20 000 IS6110 copies, seen in 45% of patients) was independently associated with a higher likelihood of incident TB infection measured by QuantiFERON (Qiagen Diagnostics, Hilden, Germany) conversion (OR, 3.2; 95% CI, 1.26–8.12) [110].
*Inconsistent correlation between sputum microscopy and newer tools*: Sputum or aerosol smear microscopy can also be graded on a semi-quantitative scale. Three studies found higher smear grade was associated with cough aerosol positivity [45, 106, 108]. Higher smear grade was associated with higher aerosol CFU [44, 106]. However, other CASS studies identified no significant associations between culturable cough aerosols and sputum smear grades [40, 107, 111].Face-mask sampling studies from South Africa showed poor correlation between culturable *M. tuberculosis* from FMS (86%) and sputum smear positivity (21%) [41, 110].

#### Clinical Measures

*Cough*: *Cough frequency and severity is associated with increased transmission risk.* Several studies suggest that cough frequency and severity may increase transmission risk. Index patient infectivity, measured by contact TST or IGRA conversion, was associated with increased cough frequency; cough frequency has also been associated with larger cavities on chest imaging [36, 37]. Cough severity was also found to be associated with high-transmission households, where more than 70% of contacts have TST conversion greater than 10 mm [38]. Although cough count in these studies was not found to be associated with TST conversion, cough frequency decreased with treatment [37, 112].
*Cavitary lung disease*: *The presence of cavitary lesions on chest imaging is associated with higher transmission risk.* Cavitary pulmonary lesions have been linked to a greater likelihood of sputum smear positivity and higher sputum bacillary load [45, 113]. The larger the cavities and the closer they are to airways, the higher a patient's cough frequency [37, 114]. Cavities may increase transmission by serving as a niche within the infected lung parenchyma with a particularly high bacillary load, and one which is resistant to the penetration of anti-TB medication. They may also promote coughing, which could, in turn, lead to transmission [39]. Contact-tracing studies have demonstrated the link between cavitary lesions and a higher risk of TB transmission to contacts [38], and this has been corroborated in a subsequent meta-analysis [39]. More recently, a Brazilian cohort study evaluating the association between Xpert Ct values and transmission showed that cavitary lesions were also an independent predictor of transmission risk (OR, 2.3) [35].

#### Effect of Treatment on Transmission Risk

While the importance of rapidly initiating effective treatment to prevent transmission is accepted, the duration of effective treatment after which patients can be deemed noninfectious remains uncertain and controversial [78, 85, 115]. Within available studies that evaluated the impact of treatment, effective treatment has been defined based on usage of multidrug regimens to which the organism is susceptible. Most studies provide data at early points in time (24–72 hours) following treatment initiation, or at longer durations (eg, 2 weeks), but data at interval durations have not been systematically evaluated. Overall, the evidence for a rapid impact of treatment on transmission comes from in vitro laboratory studies, human-to-guinea pig transmission studies, as well as cohort and clinical studies.

Early bactericidal activity studies demonstrate the rapid bactericidal effect of TB treatment. Viability of *M. tuberculosis* in sputum cultures declines substantially (eg, quantitative bacteriologic studies suggest a 90% decline) within the first 2 days of treatment with combination regimens including isoniazid, rifampin, and streptomycin [42, 43].

Culture-positivity from cough aerosols is also higher in patients prior to ATT and decreases after treatment initiation. Studies from Uganda show cough-generated aerosol cultures were positive in 25% of patients with smear-positive pulmonary TB, with a rapid decrease in the first 3 weeks of ATT. Culture-positivity in cough aerosol was associated with the lack of ATT in the prior week and was associated with fewer days of ATT [107, 108]. Other data show 30–40% of PWTB who produce cough aerosols rapidly become non-culturable after ATT initiation [44]. Culturable *M. tuberculosis* decreased from 53% of patients with drug susceptible tuberculosis (DS-TB) on less than 48 hours of ATT to 26% after 48 hours of treatment [45].

Another line of evidence for the rapid effect of ATT on transmissibility stems from studies of gene expression. Transcriptomic studies demonstrate changes in *M. tuberculosis* transcriptional profiles that may lower pathogenicity and infectiousness within days after treatment initiation. Gene expression studies using *M. tuberculosis* isolates from patients in Uganda with DS-TB demonstrate that bacterial mRNA abundance declined by more than 98% within 4 days, indicating rapid killing [53]. When *M. tuberculosis* bacilli are exposed to sterilizing TB drugs, studies have found that the organism exhibits transcriptomic changes that may impact pathogenicity and virulence from as early as 1 day after treatment initiation, despite viability and detection in mycobacterial cultures [52]. Illumina sequencing to assess the mRNA transcriptional signature of *M. tuberculosis* on 1, 3, 5, 7, and 14 days of treatment with *M. tuberculosis* transcription pretreatment revealed downregulation of genes that are associated with bacterial persistence with specific drug-induced changes noted from day 3e onwards [52].

Precursor rRNA measurement demonstrates that sterilizing drugs, such as bedaquiline and pyrazinamide, and regimens containing these drugs have a potent suppressive effect on *M. tuberculosis* rRNA synthesis (RS), in contrast to non-sterilizing drugs or weaker regimens [54]. Importantly, measurement of the RS ratio is distinct from measures of bacterial burden and drugs affect these measures differently. While suppression of the RS ratio occurred within hours of exposure to bedaquiline in vitro, indicating near-cessation of RS, CFU did not decrease substantially until 8–12 days, and results for pyrazinamide were similar [54].

Beyond laboratory-based evaluation of treatment effect, experimental human-to-guinea pig transmission model studies also consistently demonstrate the rapid effect of ATT on transmission. Human-to-guinea pig transmission studies conducted in the United States, Peru, and South Africa demonstrate that the infectiousness of people with pulmonary TB can be measured by transmission to guinea pigs. These studies demonstrate that there is wide variability in infectiousness among untreated patients [48, 49, 51, 116]. Data from a series of 1950–1960s human-to-guinea pig transmission studies in the United States and more recently in South Africa indicate that transmission may cease within 2 days of the initiation of effective ATT, irrespective of sputum bacteriologic status [47, 48, 51].

Compared with untreated patients, patients with drug-susceptible TB who were admitted to the facility at the time of initiating treatment with isoniazid, streptomycin, and PAS were 98% less likely to transmit, suggesting a rapid and almost immediate effect [47]. Another human-to-guinea pig transmission study on a ward where patients with TB and human immunodeficiency virus (HIV) coinfection were admitted for diagnosis and standard treatment in Lima, Peru, demonstrated that 90% of transmission arose from patients with TB and HIV with inadequately treated drug-resistant TB (DR-TB) rather than those with DS-TB [116]. A series of retrospective analyses of human-to-guinea pig transmission studies in patients with DR-TB in South Africa found that only 1% of guinea pigs developed infection after 3 months of exposure to 27 patients with multidrug-resistant TB who were initiated on effective ATT at the time of entry to the facility [51]. More recent unpublished human-to-guinea pig studies in South Africa demonstrate the rapid decrease in infectiousness of the BPaL (bedaquiline, pretomanid, linezolid) regimen (zero guinea pigs infected after exposure to patients who had 72 hours of treatment) compared with DR-TB regimens including bedaquiline/linezolid (no difference in guinea pig infections), suggesting a differential effect of certain drugs in the regimen (including pretomanid) on infectiousness [50].

Clinical studies also support a rapid decline in infectiousness and indicate that smear status is a poor predictor of transmission risk after treatment initiation. Data from the Madras RCT conducted in the 1950s demonstrated similar rates of household contact transmission regardless of a PWTB's sputum smear status when comparing those initiated on ATT at home (ie, ongoing [re]-exposure to household contacts) versus those hospitalized in a sanatorium for 12 months. This suggests a rapid effect of ATT without increased incremental household risk from PWTB despite ongoing exposure [15]. A prospective study evaluated TB infection in contacts of 21 PWTB who were discharged home from Cincinnati General Hospital after 2 weeks of ATT: 90% remained smear-positive, 76% with cavitary disease. The study found that 97% of contacts (72/74) who were TST negative at baseline did not convert to positive [56]. A retrospective cohort study from Arkansas showed that there was no difference in TB infection in contacts exposed to index patients who were smear positive versus smear negative (TST >9 mm 40% vs 38%) [55]. While PWTB in these studies were on treatment for 2 weeks prior to being discharged home, and these studies were nonrandomized which could lead to selection bias, these data suggest that infection risk after ATT initiation is low and that persistent smear positivity after ATT initiation does not alter contacts’ risk of TB infection [55, 56, 117]. This finding is important given that a systematic review reported that smear and culture conversion often happens after 2 weeks [46]. Although smear and culture conversion are widely used to assess treatment response, available data do not support these being effective markers of infectiousness for PWTB who are on effective ATT [57].

### Conclusions

There is a heterogeneity in infectiousness among PWTB. Prior to ATT, sputum smear microscopy, presence or absence of cough and cavities, and Xpert Ct may assist with risk prediction (ie, individuals with cough, cavitation on imaging, and sputum smear positivity or lower Xpert Ct may have higher infectious risk). Newer tools are emerging, such as cough aerosol and FMS, and may be better risk predictors than traditional sputum-based tests but are not clinically available. The exact duration of infectiousness after ATT initiation has not been defined and likely varies between individuals. However, data from EBA studies, experimental human-to-guinea pig studies, clinical studies, and transcriptomic studies suggest a rapid and steady impact of ATT (ie, within 2–4 days). After treatment initiation, sputum smear and culture status are poor predictors of infectiousness.

## 9 RECOMMENDATIONS

There was consideration of both community benefits and potential harms of interventions that place limits on individual movement or personal freedoms. The approach to recommendation development included a stepwise delineation of important decision-making principles, defining a set of possible intervention recommendations, assessment of important determinants of infectiousness and community risk, and construction of a framework by which individualized decisions could be reached. A summary of recommendations is shown in Table 1.

*Recommendation 1—Goals of respiratory isolation and restrictions*

- *Recommendation 1.1*—The decision to recommend TB respiratory isolation and restriction should consider the potential benefits and harm for both the community and the PWTB.

The legal basis for restriction of individual rights within community settings requires balancing constitutionally protected liberties with public health benefit and applicable laws. A formal recommendation was developed to ensure that public health decisions are in accordance with US law and other principles of protecting human rights and minimizing harm to PWTB [1]. The decision to recommend RIR should consider the anticipated community benefits, recognizing that TB transmission from a PWTB to another individual is a multifactorial probabilistic event based on a variety of factors that are not uniformly measurable or predictable. The intent of community-based RIR is to reduce TB transmission in the community but may result in negative consequences for PWTB in terms of effect on financial security, housing and food security, mental health, and stigma. Community-based RIR decisions should incorporate an assessment of anticipated reductions to TB transmission in the community, along with consideration of impact for PWTB.

*Recommendation 2—Defining respiratory isolation and restrictions*

- *Recommendation 2.1—*Respiratory isolation and restrictions in community settings should be conceptualized as a spectrum of tailored restrictions that are individualized for specific circumstances (see Table 2 for a suggested framework).

Community-based restrictions to human mobility or liberty should not be considered as a single uniform set of recommendations applicable to all situations. Consequently, a general “framework of restrictions” is proposed to replace singularly or narrowly defined “isolation” interventions (Table 2). Conceptualizing community-based RIR as a spectrum of possible restrictions should allow for a better balance between population benefits and the needs of the PWTB. Individual restrictions may not fit neatly into listed categories in Table 2, but these strata are a suggested framework to assist in tailored decision-making. The specific level of RIR chosen should balance the anticipated community benefits of preventing TB transmission while mitigating individual harm (Recommendation 1.1).

Determining the level of RIR is discussed further in Recommendation 5. The higher the potential for TB transmission (eg, higher individual infectious-potential TB and high-risk community settings, as outlined in Recommendation 3), the more likely an individual is to be recommended for a higher degree or duration of RIR. Nonetheless, in most instances, extensive restriction should be avoided if other risk-mitigation strategies outlined in the moderate/midlevel restriction section can be implemented. Individuals with pulmonary TB who have not yet started effective ATT may warrant more extensive restrictions. In most circumstances, the location of RIR is preferred to be in the home over hospital settings.

When indicated (see Recommendations 3–5), extensive restriction is the most restrictive form of RIR and would consist of a set of recommendations in which individuals’ movement is limited to an agreed-upon location physically separated from others and avoids shared ventilation with previously unexposed individuals (ie, community-based respiratory isolation). The location may include the individual's home or residence or, if a community setting is unavailable, a hospital airborne-infection isolation (AII) room. When extensive restrictions are implemented, any specific exceptions to recommendations should be discussed by the local public health authority and the PWTB using a patient-centered approach. All individuals should have adequate provisions for activities of daily living, such as access to healthcare, housing, and food, with support from public health departments when appropriate or needed.

Tuberculosis transmission depends on multiple factors and not all activities are anticipated to have similar transmission risk [59, 118]. In most instances, midlevel (ie, moderate) restrictions are expected to meet public health goals. Midlevel (ie, moderate) restrictions allow for some movement outside of the agreed-upon primary location of RIR for activities in settings at lower risk of transmission. For example, there is differential transmission risk based on setting and ventilation. Data from TB outbreaks have shown that living in shared indoor airspace or shared closed-ventilation systems with an individual with infectious TB has a higher risk of transmission compared with casual contact in other settings [119, 120]. Outdoor settings, locations with natural ventilation (eg, open windows), and/or UV light (to which *M. tuberculosis* is susceptible) are anticipated to reduce exposure to infectious particles generated from PWTB, with a lower probability of TB transmission to contacts [121–123]. Most outdoor activities would consequently be permissible within the context of midlevel restrictions.

Other factors that influence the likelihood of transmission from a PWTB to an exposed contact include duration, frequency, and proximity of exposures. These aspects should be considered when implementing midlevel restrictions, particularly indoor activities. There is no minimum duration or proximity of exposures that defines a likelihood of transmission. Although short durations of exposure can lead to *M. tuberculosis* infection, many contacts are not infected even after long (weeks or months) and intensive exposures [124]. Studies have further shown a higher proportion of TB infection among close contacts compared with casual contacts (ie, shorter duration and lower frequency). One set of criteria suggested in prior contact investigation guidelines and algorithms suggests a threshold of 120 hours of exposure per month to stratify transmission risk associated with exposure [124, 125]. Eight hours of exposure in a closed space has also been suggested as a threshold for contact investigations based upon evidence derived from air travel [126, 127].

Overall, lower-risk activities that result in only transient exposures (eg, less than a few [1–3] hours) in well-ventilated indoor spaces for essential activities (eg, grocery shopping for food security) may be considered as part of midlevel restrictions. Masks (surgical or KN95 or N95 if available) can be used for persons with infectious TB in indoor spaces to reduce aerosol dispersion and transmission risk, particularly if coming into contact with previously unexposed individuals [128, 129]. A study using the human-to-guinea pig transmission model demonstrated a 56% (95% CI, 33–70.5%) decreased risk of TB transmission when patients wore surgical masks [129]. More recent data from the COVID-19 pandemic have demonstrated that well-fitting masks protect against SARS-CoV-2 infection; N95 or KN95 respirators offer the best protection [130]. These data can be applied for TB transmission prevention, potentially including scenarios where there are concerns for ongoing infectiousness during ongoing treatment [95]. Individuals with infectious TB should avoid contact with individuals with an increased vulnerability to TB disease (including children under age 5 or immunosuppressed populations), and the visitation of these contacts should preferentially take place in outdoor or well-ventilated space with additional risk-mitigation strategies.

At the other end of the spectrum of RIR is a recommendation for no restrictions. As discussed in Recommendations 3–5, community-based RIRs are not indicated for most individuals considered to be noninfectious or with a low likelihood of infectiousness.

*Recommendation 3: Determining infectiousness and transmission risk*

- *Recommendation 3.1*—Prior to effective treatment initiation, PWTB with higher respiratory bacterial burden (ie, sputum smear and/or NAAT positivity, cavitation on chest imaging) may be considered as relatively more infectious than those with lower bacterial burden, with individual variability (Strong Recommendation, Moderate Certainty of Evidence).
- *Recommendation 3.2*—PWTB on less than 5 days of effective treatment should be considered relatively more infectious than those on longer durations of effective therapy (Figure 1, chart A) (Strong Recommendation, Moderate Certainty of Evidence).
- *Recommendation 3.3*—PWTB on effective treatment for at least 5 days should be considered noninfectious or with a low likelihood of infectiousness, regardless of sputum bacteriologic status during treatment (ie, smear microscopy, NAAT, or culture status), with certain exceptions (Conditional Recommendation, Moderate Certainty of Evidence).
- *Recommendation 3.4*—Overall risk of transmission to others should consider both a PWTB's infectiousness, as well as other factors including the environment of potential exposures, proximity, frequency, and durations of exposure, and biological susceptibility of contacts (Figure 1, chart B).

All PWTB should receive an assessment for the presence of pulmonary TB or TB of the respiratory tract, irrespective of initial site of TB diagnosis. This assessment may include, but is not limited to, sputum bacteriologic studies and chest imaging. As outlined in Recommendation 4 below, individuals with localized extrapulmonary TB are considered noninfectious and not recommended for RIR. Children under 10 are generally regarded as noninfectious as well. This assessment stems from the fact that most children do not have cough or aerosol of sufficient force needed for transmission, often have limited pulmonary or airway involvement, and often do not produce sputum or have low bacterial burdens within endobronchial secretions. However, children presenting with pulmonary or laryngeal TB with positive sputum bacteriologic testing (such as smear or NAAT) or cavitary disease on radiography may be considered as having adult-type TB and may be regarded as potentially infectious.

Among individuals with pulmonary TB (or TB of the respiratory tract, including laryngeal TB), an assessment for individual infectiousness should be considered a first step in the decision-making process. A full description of the evidence from the ESG's scientific review that guided guideline development is found in Section 8: Evidence Summary, with relevant data summarized below.

Prior to treatment initiation, respiratory bacterial burden and degree and strength of aerosolization may be associated with an individual's infectious capacity, as depicted on the *y*-axis of Figure 1; however, no single measurement or test reliably predicts this degree of infectiousness. For example, sputum AFB smear microscopy is commonly utilized as a measure of bacterial burden. Factors including sputum viscosity and cough strength may all alter the likelihood of generating infectious droplets. Three studies have found that higher smear grade in sputa was associated with cough aerosol TB detection, but these findings have not been universal [44, 106, 111]. At least 1 other study identified no significant associations between the ability to culture *M. tuberculosis* from aerosols generated during coughing and sputum smear microscopy [107]. Nonetheless, molecular fingerprinting studies in the United States have suggested that only 17% of transmission occurred among contacts of individuals with sputum smear–negative TB, with a relative transmission rate of 0.22 comparing individuals with smear-negative compared with smear-positive TB [101]. Other measures of bacterial burden that have been studied have included Xpert MTB/RIF (Cepheid, Inc, Sunnyvale, California, USA) Ct values, in which a lower Ct corresponds to detection of a greater amount of *M. tuberculosis* nucleic acid. While not routinely available in most clinical laboratories, higher Ct values correspond to an increased time to culture positivity and presumably lower bacterial burden [104, 105]. Other measurement techniques, including CASS and FMS, may correlate better with infectiousness than sputum-based bacteriologic studies but are not available for clinical use [110, 111, 131]. Cavitation on chest imaging is another measurement tool that is associated with greater sputum bacterial load and may be associated with increased infectiousness [45, 113].

Each of these factors may be considered when assessing the initial bacterial burden and likelihood of generating infectious aerosols in an individual who has not yet started effective therapy (Figure 1, chart A; Recommendation 3.1). Individuals are expected to be at their highest infectious potential prior to ATT initiation. Transmission risk is ultimately multifactorial. As noted in Recommendation 4, RIR may be indicated for persons with higher TB infectious potential and higher transmission risk to others (Figure 1, chart B) based on duration, proximity, frequency of exposure, and setting.

The body of evidence consistently suggests that effective ATT reduces infectiousness and is the key determinant of transmissibility; individuals not on effective treatment are anticipated to be more infectious than those who have initiated treatment (Recommendation 3.2) [59].

The viability of *M. tuberculosis* in sputum and in cough aerosols declines substantially (eg, quantitative bacteriologic studies suggest a 90% decline) within the first 2 days of ATT [42–45]. When the *M. tuberculosis* bacilli are exposed to sterilizing TB drugs, studies have found that the organism exhibits transcriptomic changes that may impact pathogenicity and virulence, despite viability and detection in mycobacterial cultures [52, 53]. Consequently, the damage done to the organisms by effective treatment likely reduces transmissibility earlier than sputum smear or culture conversion during treatment. Requirements for smear or culture conversion may therefore unnecessarily prolong community-based RIR.

Several cohort studies and trials, including some conducted in individuals with continued sputum smear positivity, provide further evidence that ATT substantially reduces infectiousness or renders individuals as noninfectious [15, 55, 56, 132]. There is additionally a lack of case reports or clinical documentation of transmission that has occurred after treatment initiation [57]. Once on treatment, laboratory bacteriologic studies may therefore not correspond to infectious potential. Visualization on sputum smear microscopy or detection in laboratory culture assays may not equate to infectiousness in aerosolized droplet nuclei (eg, bacilli visualized on microscopy or growing in culture may not be infectious) [53]; consequently, recommendations on determining infectiousness center on ensuring ongoing effective ATT with less emphasis on bacteriologic status during ongoing treatment.

The specific duration of time on effective ATT (defined as multidrug therapy to which the bacterium is susceptible) that renders a person noninfectious or with a low likelihood of infectiousness is less certain, and there may be individual variability [59]. While data across studies consistently support rapid and steady decline in transmissibility during effective treatment, not all time points have been specifically or systematically evaluated; the majority of available studies provide insights early in treatment (<72 hours) or at later durations (eg, 2 weeks).

Nonetheless, bacteriologic studies and experimental data have found that infectiousness drops rapidly, and likely within 48–72 hours, of effective ATT initiation for both drug-sensitive and drug-resistant TB, with steady declines with ongoing treatment [48, 133, 134]. Overall, the data suggest that PWTB on effective treatment are unlikely to be infectious, irrespective of sputum smear microscopy and culture status collected while on therapy.

Based on the available evidence, 5 days of ATT is the recommended duration in which the majority of persons with pulmonary TB are anticipated to be noninfectious or of low infectious potential (Recommendation 3.3), with individual exceptions. This time frame is recommended based on pragmatic consideration of individual variability, time required for clinical and public health assessment, assessment of ATT adherence and tolerability, and rapid molecular drug-susceptibility testing (when available), in conjunction with the evidence on early effect (ie, within 48–72 hours) of ATT on transmissibility.

Additional factors that clinicians may consider in assessing the level of infectiousness while on ATT include the initial bacterial load (eg, high initial bacterial burden) and site of disease (eg, laryngeal), adequacy of treatment regimens (bactericidal and sterilizing potential; drug susceptibility), and adherence and clinical response to treatment [59].

As noted above, evidence suggests that bacteriologic studies of sputum (eg, smear microscopy, NAAT, culture) collected while on effective ATT do not reliably predict post-treatment infectiousness. As such, routine collection of such bacteriologic tests solely for the assessment of infectiousness is discouraged. Clinicians may still consider microbiological response to treatment to guide clinical decisions on ATT regimen and duration (eg, 2-month culture conversion, end-of-treatment assessment of microbiological cure), but such testing has limited impact on RIR decisions.

Treatment assumes clinical expectation of effectiveness based on available phenotypic or molecular drug-susceptibility testing results, or clinical assessment. There are existing challenges to determine the initial selection of appropriate TB treatment regimens given limited accessibility to rapid drug-susceptibility testing in many US settings. Greater availability of rapid drug-susceptibility testing across the United States would assist in ensuring effective ATT, thereby improving patient outcomes and serving as a public health intervention to promptly reduce infectiousness.

In some instances where rapid drug-susceptibility testing (to at least rifamycin) is unavailable, determinations of effectiveness of therapy require assessment of the pre-test probability of drug resistance, and/or assessment of clinical response to treatment. These are many of the same considerations when constructing an initial TB treatment regimen. Appropriateness of the ATT regimen may also be assessed based on susceptibility testing of identified contacts with TB disease, initial response to treatment, or based on knowledge of local and global drug-susceptibility patterns. In such situations, clinicians may also consider whether the individual acquired TB in an area with a high incidence of drug-resistant TB, had contact with an individual with known drug-resistant TB, or has demonstrated poor clinical or bacteriologic response to empiric ATT. Notably, available data suggest that the infectiousness of persons with drug-resistant TB is anticipated to decrease rapidly with current regimens for DR-TB treatment.

Decisions on community-based RIR must consider both the individual's infectiousness (Recommendations 3.1–3.3) as well as overall risk of TB transmission to others (Recommendation 3.4). The net transmission potential within community settings requires a series of probabilistic events that may be influenced by the duration, proximity, and frequency of exposures as discussed previously, along with the setting (eg, ventilation) and biological susceptibility to infection of an individual exposed to infectious aerosols. As noted earlier, ventilation and air circulation are important determinants of infectious risk, with less likelihood of transmission in outdoor, well-ventilated environments [112, 119, 120, 135–137]. Transmission is more likely to occur with repeated, long durations (eg, multiple hours) of contact in close proximity to a PWTB, compared with more brief exposures.

These factors can be taken together, along with an individual's relative infectiousness, to determine the overall risk of transmission to occur in community settings (Figure 1, chart B; Recommendation 3.4) in order to make determinations regarding RIR (Recommendation 4).

*Recommendation 4—Determining whether community-based RIR is indicated (Table 3)*

- *Recommendation 4.1—*RIR is not recommended for persons with noninfectious forms of TB (ie, localized extrapulmonary TB without pulmonary involvement, as confirmed by sputum bacteriologic studies and/or chest imaging).
- *Recommendation 4.2—*People with pulmonary TB on effective treatment and with a low likelihood of infectiousness should not have restrictions in most circumstances (ie, RIR should be removed, if present), with individual exceptions for situations involving higher-risk community settings and populations (eg, children <5 years, immunosuppressed individuals) (Conditional Recommendation, Moderate Certainty of Evidence).
- *Recommendation 4.3—*Community-based RIR may be considered for PWTB who have higher infectious potential in which there is judged to be higher risk of transmission to the community (Conditional Recommendation, Low Certainty of Evidence).

Initial decisions for community-based isolation and restrictions (Table 3) should consider the PWTB's infectiousness (Figure 1, chart A) and the anticipated net risk of community-based transmission (Figure 1, chart B), and should balance potential harms to the individual and community benefits (Recommendation 1).

Individuals without pulmonary TB are not expected to pose an infectious risk to others in the community and community-based RIR is not recommended (Recommendation 4.1).

Alternatively, individuals with pulmonary TB are expected to be most infectious prior to ATT initiation, with steady declines in infectiousness with effective treatment. Those who have not yet completed effective ATT for at least 5 days are expected to have a higher infectious potential than those on longer durations of treatment (Recommendation 3.3). Community-based RIR should be considered for PWTB with higher infectious potential who have not completed at least 5 days of effective ATT (Recommendation 4.3). The degree and type of RIR are discussed in Recommendation 5.

Community-based RIR is not recommended (and can be discontinued if implemented) for most individuals on at least 5 days of effective ATT who have low infectious potential, with some exceptions (Recommendation 4.2). Five days of effective ATT as a threshold for removal of community-based RIR for most PWTB stems from considerations of available evidence of rapid treatment impact on infectiousness, while also balancing considerations of potential harms to PWTB from prolonged restrictions.

There may be individual exceptions to extend the duration of RIR when there is concern for prolonged infectiousness (eg, high pretreatment bacterial burden; additional clinical or laboratory evaluation to assess effectiveness is needed; concerns for adherence or tolerability) and/or when the community risk or consequence of transmission is deemed high (eg, higher-risk settings [crowded, poor ventilation]; settings with vulnerable populations, including children <5 years or immunosuppressed individuals).

Examples of situations that could warrant implementation or continuation of RIR beyond 5 days of effective ATT may include the following: scenarios of confirmed or suspected drug-resistant TB in which there is ongoing clinical or laboratory evaluation to determine the effectiveness of the treatment regimen, high pretreatment bacterial burden and anticipated activities or exposures at higher risk of TB transmission (eg, prolonged, frequent, close contact to previously unexposed individuals in poorly ventilated and/or indoor environments), or potential exposures of vulnerable populations, including but not restricted to children younger than 5 years or immunosuppressed persons (healthcare settings) who have a higher risk of TB infection and progression to TB disease.

The optimal duration of RIR extensions in such situations where PWTB are on appropriate therapy is uncertain. Longer durations of ATT (eg, 14 days) may reduce infectious risk even further (Recommendation 3.2), but there is no specific duration that universally predicts that a PWTB is noninfectious; longer durations of RIR are anticipated to have increasing potential for harms to the PWTB, with diminishing returns in terms of preventing community transmission. Scenarios in which RIR is prolonged while on effective ATT (eg, >14 days) may warrant additional expert consultation and efforts to mitigate community and patient harm, particularly for marginalized populations.

*Recommendation 5—Determining Level of RIR*

- *Recommendation 5.1—*When considering restrictions for PWTB, a moderate or midlevel range of RIR should be considered appropriate (Table 2) in most circumstances, with individual exceptions.
- *Recommendation 5.2—*Specific RIR levels (eg, low, moderate, or extensive) (Table 2) and duration for PWTB should be reassessed routinely (at least weekly) and may be modified based on individual considerations or changing circumstances.
- *Recommendation 5.3—*When RIR is implemented, support should be provided to patients to mitigate anticipated and experienced harms.

When a PWTB is considered to have a relatively higher degree of infectiousness (ie, as outlined in Recommendation 3 and Figure 1), and there is a determination that RIR is indicated (Recommendation 4) based on weighing benefits and harms to the community and individual, the least-restrictive RIR provisions should be recommended that achieve public health goals.

In most such instances where community-based RIR is indicated, a moderate or midlevel of isolation restriction (Table 2) is likely to be appropriate. The public health department should identify a location for community-based RIR that minimizes new exposures, particularly during periods of highest infectiousness (eg, prior to effective treatment), using a patient-centered approach. Infectious PWTB should stay at the agreed-upon location whenever possible. Moderate restrictions would allow for most outdoor activities, recognizing that transmission risk is lower in outdoor, well-ventilated environments (particularly activities that avoid prolonged close contact with previously unexposed individuals).

Once effective ATT has been initiated, and during the period where infectiousness is expected to be declining but RIR is still recommended, some essential or high-priority indoor activities may be considered based on necessity (eg, ensuring food security, healthcare) and balancing community and individual risks and benefits; wearing of surgical masks by PTWB may further reduce infectious potential and such risk-mitigation strategies can be considered as part of moderate restrictions.

Extensive restrictions (Table 2) may be considered in specific circumstances involving PWTB with higher infectious potential and higher community transmission risks. Individuals are expected to have their highest infectious potential prior to starting ATT, and individuals with high pretreatment bacterial burden may be particularly infectious. The risk of transmission of known drug-resistant TB may also confer greater community harm. Some individuals may not agree or be unable to agree to the provisions of lesser degrees of restrictions (ie, agree to avoid indoor, poorly ventilated settings; avoid contact with previously unexposed vulnerable populations). In such situations, a more extensive set of restrictions with fewer provisions for movement outside of an agreed-upon location may be considered.

The duration of any restrictions should be reassessed at regular intervals (eg, weekly) and consider the ongoing degree of infectiousness, adherence and response to ATT, anticipated community risk of TB transmission, and impact on the individual (Recommendation 5.2). While the duration of RIR is anticipated to be less than 1 week in most instances where PWTB have initiated effective ATT, individual exceptions where there is a high risk or consequence of community TB transmission may warrant tailored extensions (Recommendations 4.2 and 4.3).

These durations are not clearly defined, as they require individual assessment of infectiousness and the risk of community TB transmission. Most individuals, including those with high initial pretreatment bacterial burden (ie, smear-positive and cavitary disease), are expected to have low infectious potential after completing 5 days of ATT and progressively lower infectiousness after 10 to 14 days; alternatively, longer durations of RIR are anticipated to increase the potential for individual harms to PWTB. In scenarios where RIR has been prolonged beyond 14 days, additional expert consultation should be considered to balance community benefits and individual harms and ensure legal due process. Recognizing that community-based RIR may limit individual freedoms, and have a negative impact on housing, food security, and financial security, public health programs should consider providing support to PWTB as resources allow.

## 10 CONSIDERATIONS FOR IMPLEMENTATION

There are several factors to consider when implementing these recommendations. These guidelines were developed by the NTCA and key stakeholders based on the available scientific evidence and considerations of ethical principles in public health policy but are not legally binding. There may be situations where the recommendations outlined in the guidelines do not align with state and local laws. These guidelines serve as a framework to help public health programs advocate for changes and updates to state and local laws and policies. While not explicitly discussed in these guidelines, existing legal guidance suggests there should be consideration for substantive and legal due process when implementing TB RIR [1]. Public health authorities are recommended to balance population benefits of the potential to reduce TB transmission and individual harms from restricting movement and other liberties [23]. A summary of key take-home points to aid implementation of community-based guidelines is shown in Figure 2.

Among the goals of the guideline development process was to incorporate a patient-centered ethics-based framework into community-based RIR decisions, to balance considerations for individual and public health. To that end, implementation of these guidelines should strive to respect and consider a PWTB's autonomy and liberty, while also assessing community risks of transmission. Decisions related to community-based RIR are anticipated to be a dynamic process. Community-based RIR should be reassessed as new information, including evidence of harm to the PWTB, is identified. Other considerations may include evolving community context or anticipated risks, information on drug susceptibility, considerations of ATT adherence, and longitudinal assessment of infectiousness. Public health practitioners and clinician decision-makers should consider the potential for implicit and explicit bias that may influence decision-making.

The recommendations made in these guidelines acknowledge that treatment efficacy is a key determinant of infectiousness of a PWTB. However, the practical availability of rapid drug-susceptibility testing is limited in many parts of the United States. Nonetheless, clinical decisions on initial ATT selection may proceed based on pre-test probability of drug resistance based on epidemiologic considerations. When there are increased concerns for the appropriateness of an ATT regimen (eg, rifamycin resistance detected on Xpert MTB/RIF, or contact with an individual with known drug-resistant TB), programs and clinicians are encouraged to seek out rapid molecular drug-susceptibility testing through local and state public health laboratories, or through the CDC's Molecular Detection of Drug Resistance (MDDR) service. When rapid drug-susceptibility testing is not readily available, clinical and/or microbiological response may also provide insights into the effectiveness of a selected ATT regimen. Expert consultation should be sought if there are concerns for drug resistance or concerns regarding the effectiveness of the treatment regimen.

It is common to make an initial diagnosis of TB in hospital or healthcare settings. In most instances where RIR are needed, persons can be discharged to home or community settings if the person is not acutely ill, particularly once effective ATT has been initiated. Home-based care and RIR is preferred, when possible, over hospital-based AII. Public health professionals should ensure that there is stable housing, and there will not be new household exposures to individuals not previously in contact with the PWTB when assessing appropriateness of transition from healthcare to community-based settings. Where possible, PWTB who are considered infectious according to Recommendation 3 (Determining infectiousness) should minimize or avoid contact with immunologically vulnerable individuals or populations, including children less than 5 years or immunosuppressed persons.

A consolidated step-by-step guide to facilitate implementation of the recommendations in this guideline for persons newly diagnosed with TB is provided in Table 4 (initial determination of RIR). The decision to de-isolate after ATT has commenced is summarized in Table 5. The available evidence suggests that the damage done to the mycobacterium with effective ATT rapidly reduces infectiousness, before treatment effects are evident on laboratory tests such as mycobacterial culture, smear microscopy, or NAAT testing. A pragmatic approach to community-based RIR therefore focuses on the provision of effective ATT and does not depend upon sputum smear or culture conversion. While there is no definitive duration that reliably predicts noninfectiousness, 5 days of ATT is expected to reduce infectivity to low levels in most instances. Infectivity is expected to fall even further to negligible levels even in those with high initial bacterial burden as treatment progresses (eg, 14 days). As such, community-based RIR is expected to be discontinued by 5 days in most instances, with extensions in individualized circumstances where there is deemed to be higher community risks of transmission. When community-based RIR extends past 14 days, further expert consultation and review are warranted.

## 11 KNOWLEDGE GAPS AND FUTURE RESEARCH DIRECTIONS

These guidelines have limitations based on areas of scientific knowledge gaps. Implementation may be affected based on existing local practices and laws. Conducting research to understand the feasibility, acceptability, reach, and impact of the updated recommendations is encouraged and will help to inform future guideline updates.

Additional research into the infectivity of TB would inform future guideline updates to refine the appropriate duration of community-based RIR. In the absence of clear biomarkers, these guidelines relied on available bacteriologic studies, experimental data from human-to-guinea pig transmission models, epidemiologic studies, and expert opinion. Additional research is needed to quantify the proportion of TB transmission that is avertable by community-based RIR, acknowledging that the majority of transmission may occur prior to diagnosis and any RIR interventions. Tools to better quantify the patient and community impact of RIR interventions are needed. A suggested list of research priorities based on the guideline development process includes, but is not limited to, the following:

1. Tools or biomarkers to quantify individual infectiousness among PWTB
2. Development and implementation of rapid diagnostics for first- and second-line TB drugs
3. Tools or biomarkers to measure clinical and bacteriologic response to ATT
4. Tools to better assess community risk of TB transmission
5. Tools to better measure patient harms related to TB diagnosis, ATT, and restrictions

## Supplementary Material

ciae199_Supplementary_Data

## Supplementary Data

Supplementary materials are available at *Clinical Infectious Diseases* online. Consisting of data provided by the authors to benefit the reader, the posted materials are not copyedited and are the sole responsibility of the authors, so questions or comments should be addressed to the corresponding author.

## Notes

***Acknowledgments.*** The Guideline Development Group (GDG) acknowledges the support of the systematic review and evidence synthesis team, including Ed Nardell, Sundari Mase, Sonal Munsiff, Abarna Pearl, Amanda Biewer, Laura Young, Leonard Mukasa, Naveed Delrooz, Advaith Subramanian, and Sarah Miller. The scientific evidence that supported these guidelines is or will be presented in separate publications. Additional editorial review was provided by Jason Cummins, Elizabeth Foy, and John Bernardo, as well as the Board of Directors of the National TB Coalition of America.

***Financial support***. This work was supported by the National Institutes of Health (grant numbers NIAID K23 AI132648-05 and DP2-AI176896 to R. N. and P30AI18436 to M. S.).

***Potential conflicts of interest.*** The authors: No reported conflicts of interest. All authors have submitted the ICMJE Form for Disclosure of Potential Conflicts of Interest.
