# Supplementary material for: National Tuberculosis Coalition of America Guidelines for Respiratory Isolation and Restrictions to Reduce Transmission of Pulmonary Tuberculosis in Community Settings
Source: Clin Infect Dis. 2024 Apr 18;83(4):e684–714. doi: 10.1093/cid/ciae199 (PMC13675926; doi:10.1093/cid/ciae199)
Supplement: Supplementary file 1 [file ciae199_supplementary_data.docx]

# Appendix

## Appendix 1: Evaluation of patient harms related to community-based respiratory isolation and restriction

There are no existing, validated tools to quantify patient harm among PWTB experiencing community-based RIR. In order to inform implementation of these guidelines, a literature review was conducted to identify available tools that could be utilized by public health programs to assess patient experiences during RIR. There was a lack of data on how best to reliably measure the multiple dimensions of patient experiences during community-based RIR.

Drawing on available literature on assessment of mental health, stigma, and socio-economic factors, a selection of probing questions was curated to aid public health programs in their assessment of patient impact during community-based RIR. This list is not suggested to be comprehensive and should not be regarded as a validated scale to measure harm experienced by PWTB in the context of community-based RIR. This set of probing questions may assist programs in considering the range and spectrum of impacts experienced by PWTB during community-based RIR.

The potential for patient harm was stratified into commonly identified domains during the literature review, including mental health, stigma, and various socio-economic factors. A representative set of example questions was derived from the literature in order to provide a snapshot of the multiple different dimensions of patient experiences affected by RIR. Each person's experience of TB and community circumstances are unique and as such, this list may not encompass all potential considerations. Nonetheless, the aim of this list of dimensions and possible probing questions is to facilitate and encourage incorporation of patient impact into decision making (Recommendation 1) related to the usage of community-based RIR, and to ensure that the scope of restrictions (Recommendation 2) balances risks and benefits to the community and the patient. As the risk of negative consequences for the PWTB increases due to public health directed interventions such as community-based RIR, additional mitigation strategies, patient support, or tailoring of restrictions may be required (Recommendation 5.4).

*Examples of probing questions to assess impact and concerns of community-based RIR for PWTB*

General

- What are your feelings and thoughts about the proposed restrictions?
- Do you anticipate any challenges to following the proposed restrictions?

Stigma

- Do you feel that people may not consider you or listen to you because of TB? [1]
- Are you afraid to tell your family that you have TB? [2]
- Are you concerned that TB will affect your relationships to others in your family or community?

Housing

- Are you experiencing homelessness? [3]
- Do you feel unsafe inside your home? [3]
- Do you worry about being forced to move? [3]

Food

- In the past year were you ever hungry but did not eat because there wasn't enough money for food? [4, 5]
- In the past year could you often, sometimes, or never afford to eat balanced meals? [4, 5]
- Are you concerned about access to food as a result of your TB diagnosis or treatment plan?

Job Security

- Do you think you might lose your job in the near future? [6]
- Are you able to work remotely?
- How do you anticipate respiratory isolation and restrictions will impact your current or future employment?

Financial Security

- In the past year have you have trouble paying for: [7]
  - Rent or mortgage
  - Medical care
  - Other bills
- Have you borrowed money in the past year? [7]
- Do you have concerns about money during respiratory isolation and restrictions?

Mental Health (Additional existing tools may include but are not limited to the PHQ-9 or Beck’s depression index) [8-11]

- In the past 2 weeks, how often have you been bothered by the following problems?

(Options: a)Less than 1-2 days, b)several days, c)more than half the days, d)nearly every day, or e) not at all?) [12]

- Feeling down, depressed, or hopeless? [12]
- Feeling more irritated, grouchy, or angry than usual? [12]
- Feeling nervous, anxious, frightened, worried, or on edge? [12]
- Thoughts of actually hurting yourself? [12]
- Hearing things other people couldn’t hear, such as voices even when no one was around? [12]
- Unpleasant thoughts, urges, or images that repeatedly enter your mind? [12]
- Using drugs or drinking at least 4 drinks of any kind of alcohol in a single day? [12]

Other

- Do you have anybody to help you solve economical, familial or sentimental problems? [1]
- Will restrictions or isolation interfere with your ability to access or take your medications as normal?
- Do you feel that restrictions or isolation will affect your ability to independently complete tasks of daily living?
- Are you responsible for the caretaking of someone else which may be affected by restrictions or isolation?
- Are there any other concerns that need to be considered?

**These questions do not represent a comprehensive list of possible harms experienced by PWTB during RIR. These probing questions may be tailored or altered based on individual circumstances. Individuals answering yes to one or more items in this list may warrant considerations for additional support, re-assessment of level of RIR, or other mitigation strategies to minimize potential patient harm.*

## Appendix 2: Strength of recommendations and certainty of evidence

*Overview:* The guidelines sought to utilize the best available scientific evidence and incorporated an ethics-informed framework (described in Section 8) for recommendation development. Multiple approaches to grading strength of recommendations and certainty of evidence were considered [13-15]. Several limitations to commonly used approaches including the GRADE approach were identified. In certain instances, the GDG sought to make recommendations considered important to articulating foundational principles to respiratory isolation decisions, but not appropriate for rating the certainty of evidence. Such statements were considered to fall outside the domain of standard GRADE processes and are considered ‘good practice statements’ [16]. Recommendation 1 (Goals of RIR), Recommendation 2 (Defining RIR), Recommendation 3.4, Recommendation 4.1, and Recommendation 5 (Determining level of RIR) were considered good practice statements and did not have an associated strength of recommendation or certainty of evidence assessment.

The GRADE approach is recognized to have limitations when applied to public health guidelines [17]. Among the challenges is the need to address individual and population outcomes in the same guideline and systematic review, particularly when benefits and harms differ depending on which perspective is taken. The strength of recommendation and certainty of evidence definitions were therefore adapted from several sources including GRADE, USPTF, ACIP, and Canadian National TB Standards [14, 15, 18, 19]. “Strong recommendations” were those where there was high certainty that the net benefits were substantial and that the action or assessment would be the preferred choice for most PWTB and public health decision makers. These recommendations are usually based on “high quality” evidence. Other considerations include whether the anticipated magnitude of benefits is expected to outweigh harms in most circumstances. “Conditional recommendations” were those where there was low or moderate certainty that the net benefits outweighed harms or that there is uncertainty or variability in the anticipated benefits and harms. These recommendations are usually based on “low” or “moderate” certainty of evidence. When judging certainty of evidence, the study design, study quality, inconsistency of findings, and indirectness were considered [18, 20]. Broadly, when the evidence was based on one or more high quality randomized trials with low risks of bias and clear findings of benefits and harms directly addressing the recommendation questions, and consistent findings and effect size across studies, the certainty of evidence was considered “high”. When there were concerns regarding study design, risks of bias, or concerns for inconsistency or indirectness, the certainty of evidence was considered low or moderate [13]. Low or moderate quality suggests that further research is likely to have an important impact on the confidence in the effect estimates and may change estimates. For analytical studies, the Quality Assessment of Technical Studies was adapted [21]. The evidence was synthesized into evidence to recommendation tables, adapted from existing frameworks to guide recommendation development and are shown below [15].

The following questions were used to guide the certainty of evidence assessments for the purpose of developing the evidence to recommendation tables.

1. PICO 1: Does respiratory isolation improve or worsen outcomes among persons with pulmonary TB and the community when compared to no isolation?
   a. Outcomes:
      i. TB Infection amongst contacts
      ii. TB Incidence amongst contacts
      iii. TB mortality
      iv. TB hospitalization/duration
      v. Patient and health system costs
      vi. Mental health/stigma
2. Scoping Review Questions:
   a. Are bacteriologic studies (prior to treatment) associated with transmissibility of *M. tuberculosis* from PWTB to others?
   b. Is cough or respiratory aerosols (prior to treatment) associated with transmissibility of *M. tuberculosis* from PWTB to others?
   c. Is chest imaging (prior to treatment) associated with transmissibility of *M. tuberculosis* from PWTB to others?
   d. Does treatment reduce transmissibility of *M. tuberculosis* from PWTB to others?
      i. Are bacteriologic studies (after treatment initiation) associated with transmissibility of *M. tuberculosis* from PWTB to others?
      ii. Is cough or respiratory aerosols (after treatment initiation) associated with transmissibility of *M. tuberculosis* from PWTB to others?

*Assessing certainty of evidence*: Several domains were assessed that led to overall assessment of certainty of evidence. Study Design refers to the basic study design, broadly categorized as analytical studies, observational studies, and randomized trials [18]. Randomized trials were considered the highest certainty of evidence, while observational and analytical studies are low certainty of evidence, and any other designs were considered very low certainty of evidence. Recognizing that randomized trials are not always feasible or applicable, high quality observational and analytical studies with consistency of results, appropriate methods, and directness of evidence were also considered high quality evidence. Study quality, judged by the systematic review team, refers to the detailed study methods and execution and risks of bias. Inconsistency refers to a lack of similarity for estimate of effects across studies. Indirectness refers to the extent to which people, interventions, and outcomes measures may be unrelated to those of interest.

## Table 1: Certainty of evidence assessment for each guideline development question

| Question | Domains assessed | Certainty of Evidence assessment | Notes |
| --- | --- | --- | --- |
| PICO 1i.: Does isolation reduce TB infection among contacts? | Study Design  Quality Assessment  Inconsistency  Indirectness | Very low | A single RCT; some observational and modeling studies. Uncertain effect size. Downgraded due to limitations in study design, quality, inconsistency of findings across studies, and indirectness and applicability of studied interventions. |
| PICO 1ii.: Does isolation reduce TB incidence among contacts? |  | Very Low |  |
| PICO 1iii.: Does isolation reduce TB mortality among contacts? |  | Very Low |  |
| PICO 1iv.: Does isolation increase duration of hospitalization for PWTB? |  | Moderate | Primarily observational studies. Downgraded for study design and indirectness to current guideline intervention (community-based isolation) with most studies evaluating hospital-based isolation duration. Effect size was consistent across studies suggesting increased duration with respiratory isolation. |
| PICO 1v.: Does isolation increase patient and/or health system costs? |  | Moderate | Primarily observational studies. Downgraded for study design and indirectness to current guideline intervention (community-based isolation) with most studies evaluating hospital-based isolation with prolonged durations. Effect size was consistent across studies suggesting increased health system costs with respiratory isolation. |
| PICO 1vi.: Does isolation worsen mental health outcomes and stigma among PWTB? |  | Moderate | Primarily observational and qualitative studies. Downgraded for study design and indirectness to current guideline intervention (community-based isolation) with most studies evaluating hospital-based isolation and prolonged durations. Effect size was consistent across studies suggesting negative impact on mental health and stigma attributable to respiratory isolation. |
| Scoping A.  Are bacteriologic studies (prior to treatment) associated with transmissibility of *M. tuberculosis* from PWTB to others? | Study Design (or Experimental design for analytical studies)  Inconsistency  Indirectness  Materials and methods (for analytical studies) | Moderate | Downgraded one level for study design. One RCT with limitations in direct applicability of studied outcomes. Epidemiologic, observational studies, surveillance data. Evidence of a dose response with bacterial burden measured by sputum, FMS, cough aerosol smear-microscopy, NAAT, and culture prior to treatment. |
| Scoping B.  Is cough (prior to treatment) associated with transmissibility of *M. tuberculosis* from PWTB to others? |  | Low | Downgraded one level for study design. Primarily observational studies. Downgraded for limitations in indirectness of study outcomes (transmission) and uncertainty in effect size. |
| Scoping C.  Is chest imaging (prior to treatment) associated with transmissibility of *M. tuberculosis* from PWTB to others? |  | Very low | Downgraded one level for study design. Primarily observational studies. Downgraded for limitations in indirectness of study outcomes (transmission) and uncertainty in effect size. Limited studies with limitations in consistency of effect size measurement. |
| Scoping D.  Does treatment reduce transmissibility of *M. tuberculosis* from PWTB to others? |  | Moderate | Downgraded one level for study design. One RCT with limitations. Analytical (bacteriologic) studies, human-to-guinea pig studies assessed to be appropriate study designs to inform effects. Downgraded one level for indirectness due to uncertainty in effect size at varying durations of treatment, specific treatment regimens. Increased grade one level based on evidence of dose response and consistency of findings across study designs. The direction of effect size suggests treatment reduces transmissibility. |
| Scoping D.1.  Are bacteriologic studies (after treatment initiation) associated with transmissibility of *M. tuberculosis* from PWTB to others? |  | Moderate | Downgraded one level for study design. One RCT with limitations. Analytical (bacteriologic) studies, human-to-guinea pig studies assessed to be appropriate study designs to inform effects. Downgraded one level for indirectness due to uncertainty in effect size at varying durations of treatment, specific treatment regimens. Increased grade one level based on evidence of dose response and consistency of findings across study designs. The direction of effect size suggests bacteriologic studies after treatment initiation are not associated with transmissibility of *M. tuberculosis* from PWTB to others. |
| Scoping D.2.  Is cough (after treatment initiation) associated with transmissibility of *M. tuberculosis* from PWTB to others? |  | Very low | Downgraded two levels for study design and availability. Lack of evidence on association of cough after treatment initiation. Downgraded for consistence and indirectness of available data. |

The purpose of Evidence to Recommendation (EtR) frameworks is to help the guideline development groups move from evidence to recommendation decisions and offer transparency around the important deliberations [15].

## Table 2a: Evidence to Recommendation tables

| **Should respiratory isolation and restrictions (RIR) be implemented in community settings for persons with pulmonary TB?** | | | |
| --- | --- | --- | --- |
| **Domain** | **Signaling question** | **Working group judgement** | **Evidence summary and notes** |
| Problem | Is the problem of public health significance? | No  Probably No  Probably Yes  **Yes** | A total of 600 known TB deaths occurred in the U.S. for 2020, the highest mortality reported among persons with TB (PWTB) nationally since 2006 [22]. Details provided in Full Text of Recommendations, Section 4. Background and Rationale. |
| Benefits and harms | How substantial are the desirable anticipated effects? | Minimal  Small  Moderate  Large  **Varies**  Unknown | The beneficial effects are averted transmission of TB and are anticipated to differ based on multiple factors. These factors can include context specific variables including the proximity, duration, environment, and frequency of contact with others. The anticipated magnitude of beneficial effects from averted transmission may also depend upon the infectious potential of the PWTB. Infectious potential may further vary based upon pre-treatment disease burden and particularly with the receipt and duration of effective treatment. Overall, usage of effective treatment is anticipated to be the most important variable determining likely potential benefits from community-based RIR. Data on the proportion or magnitude of TB transmission avertable through community-based isolation is also lacking. Available evidence from contact investigations in the US suggests ~15% of evaluated contacts are identified to have TB infection. In the US ~12% of diagnosed TB are attributable to recent transmission. A large proportion of TB transmission may occur prior to diagnosis. Prior to treatment the effect size of reduced incidence from implementing community-based RIR is uncertain and is estimated to be small to moderate in most community settings. Based on evidence from one RCT, laboratory analytical studies, human-to-guinea pig transmission studies, and cohort studies, the effect sized of reduced incidence from implementing community-based RIR is uncertain and is estimated to be minimal to small. Alternatively, the anticipated harms from RIR may also vary based on individual circumstances, specific provisions of RIR, as well as duration of RIR. |
|  | How substantial are the undesirable anticipated effects? | Minimal  Small  **Moderate**  Large  Varies  Unknown | The undesirable effects of isolation on PWTB were estimated to be moderate based on individual circumstances. Available evidence consistently suggests that there are negative consequences to mental health, stigma, and financial security, and health system costs attributable to respiratory isolation. However, most data was from hospital based isolation, with less data regarding community-based isolation where these effects may differ. Undesirable effects are anticipated to be proportional to duration of isolation and available support and resources and may vary. Longer durations of isolation were anticipated to have greater undesirable effects than shorter durations. See Full Recommendations, Section 9. |
|  | Do the desirable effects outweigh the undesirable effects? | Minimal  Small  Moderate  Large  **Varies**  Unknown | Benefits may include averted TB transmission within communities. Undesirable effects may include loss of financial security, housing security, food instability, negative mental health outcomes, and increased stigma for PWTB. The relative desirable and undesirable effects are anticipated to vary based on individual infectiousness, community context, and individual considerations and resources and available support through public health services. |
|  | What is the overall certainty of the evidence for critical population outcomes (benefits)? | No studies found  **4 (very low)**  3 (low)  2 (moderate)  1 (high) | Based on the available data on transmissibility of *M. tuberculosis,* relative effect of treatment, and limited randomized trial data, there is limited certainty that implementation of community-based isolation reduces population level transmission (incident infection), incidence (TB disease), morbidity or mortality. Notably, the systematic review was not able to ascertain the relative effect size of isolation compared to other TB prevention measures including earlier diagnosis and treatment, usage of contact investigations, and LTBI diagnosis and treatment to prevent TB disease. See Full Recommendations, Section 9 |
|  | What is the overall certainty of the evidence for critical patient outcomes (harms)? | No studies found  4 (very low)  3 (low)  **2 (moderate)**  1 (high) | The available studies suggest respiratory isolation has negative consequences on mental health, stigma, individual finances and health system costs, particularly for marginalized or vulnerable populations. Limitations of available studies include study design, namely the lack of randomized trials or non-isolation control measures, applicability (hospital versus community setting), heterogeneity in isolation implementation (e.g., duration) and indirectness since isolation was rarely or primarily the intervention being evaluated. |
| Values | Do PWTB and community members feel that the desirable effects are large relative to the undesirable effects? | No  Probably no Probably yes Yes  **Varies**  Don't know | There was limited evidence on the values and preferences of PWTB and community representatives or public health practitioners related to community-based RIR. Available evidence suggested RIR has negative effects on mental health, stigma, and other patient important outcomes, particularly with prolonged isolation but may vary; the anticipated desirable effects were also considered to vary by individual and community circumstances. Included studies mentioned concerns raised by families of PWTB about hospital-acquired infection or reinfection in certain settings such as prisons. The GDG included key stakeholders and public health practitioners, clinicians, nurses, epidemiologists, and TB survivors and advocates and considered that the anticipated desirable and undesirable effects are anticipated to vary widely based on conditions and duration of RIR, individual and community resources, as well as clinical factors and community context. |
|  | Is there important uncertainty about or variability in how much people value the main outcomes | No  Probably no **Probably yes** Yes  Don’t Know | Discussions with key stakeholders and GDG members identified uncertainty and variability in how much people value the primary population versus individual outcomes. |
| Acceptability | Is the intervention acceptable to key stakeholders? | Probably no Probably yes Yes  **Varies**  Don't know | There is evidence for heterogeneity in terms of support to PWTB, duration and location of community-based RIR. The acceptability of the intervention to PWTB and members of the community is expected to vary based on specific conditions and provisions of RIR. Shorter durations in community settings with less stringent restrictions that achieve public health goals are anticipated to be more acceptable to key stakeholders than longer durations of strict restrictions. |
| Resources | Is the intervention a reasonable and efficient allocation of resources? | **Probably no** Probably yes Yes  Varies  Don't know | There is a lack of cost-effectiveness analysis to assess patient, health system, and societal costs related to the implementation of community-based RIR. |
| Equity | What would be the impact on health equity? | Reduced  **Probably reduced**  Probably no impact  Probably increased  Varies  Don't know | It was anticipated that RIR may have different levels of undesirable effects based on socioeconomic and other demographic considerations. Marginalized or economically vulnerable populations were anticipated to experience larger negative consequences from community-based RIR due to negative impacts on mental health and socioeconomic barriers to remaining isolated including loss of employment, effects on education, as well as the stigma related to RIR in the context of a TB diagnosis. |
| Feasibility | Is the intervention feasible to implement? | No  Probably No  **Probably Yes**  Yes  Varies  Don’t know | There is long standing pragmatic experience with implementation of community-based RIR. However, implementation is heterogeneous across states. Barriers to implementation include determination of infectiousness of PWTB, availability of rapid drug susceptibility testing to help guide effective treatment, and available resources to support PWTB during community-based RIR. |

Evidence to recommendation tables adapted from: ACIP Evidence to Recommendation User's Guide[15].

Given the judgement that benefits ,harms, values, and acceptability varies based on multiple factors, decisions on the certainty of evidence and strength of recommendations were made on the balance of consequences for several categories and conditions. Based on the evidence in the scoping review (see Full Recommendations, Section 9), judgements were made based on pre-treatment bacterial burden, and usage of effective treatment (as defined in Full Recommendations). Due to the lack of evidence that bacteriologic studies (i.e., sputum smear, NAAT, culture) correlate with ongoing infectiousness after initiation of effective treatment, additional stratifications based on these parameters after treatment initiation were not included.

## Table 2b: Evidence to Recommendation Table Summary Judgements on usage of community-based RIR for specific conditions.

| Should community-based RIR be used for persons with pulmonary TB with [condition]? | | |
| --- | --- | --- |
| Condition (see Table 3) | Judgements | Notes |
| Pre-Treatment, high and low bacterial burden (see Table 3) | Undesirable consequences clearly outweigh desirable consequences in most settings | Persons with pulmonary TB are anticipated to be most infectious prior to treatment initiation. The evidence suggests those with higher bacterial burden (smear-positive, NAAT-positive, cavitary disease) may be more infectious pre-treatment than those with lower bacterial burden. Nonetheless, it was judged that the desirable consequences of possible averted TB transmission in most settings probably outweighs undesirable consequences of community-based RIR, provided support is provided to mitigate harms (recommendation 5.3) for both situations of high and low bacterial burden. |
|  | Undesirable consequences probably outweigh desirable consequences in most settings |  |
|  | The balance between desirable and undesirable consequences is closely balanced or uncertain |  |
|  | **Desirable consequences probably outweigh undesirable consequences in most settings** |  |
|  | Desirable consequences clearly outweigh undesirable consequences in most settings |  |
| Treatment<= 5 days, high and low bacterial burden |  |  |
|  | Undesirable consequences clearly outweigh desirable consequences in most settings | Persons with pulmonary TB are anticipated to be most infectious prior to treatment initiation, with rapid reduction in infectiousness with effective treatment irrespective of bacteriologic studies during treatment. The specific duration of treatment at which most PWTB are non-infectious is uncertain. Available data suggests steady declines in infectiousness with longer durations of treatment.  Nonetheless, it was judged that the desirable consequences of possible averted TB transmission at less than five days of effective treatment in most settings probably outweighs undesirable consequences of community-based RIR, provided support is provided to mitigate harms (recommendation 5.3) for both situations of high and low pre-treatment bacterial burden. |
|  | Undesirable consequences probably outweigh desirable consequences in most settings |  |
|  | The balance between desirable and undesirable consequences is closely balanced or uncertain |  |
|  | **Desirable consequences probably outweigh undesirable consequences in most settings** |  |
|  | Desirable consequences clearly outweigh undesirable consequences in most settings |  |
| Treatment>= 5 days, high pre-treatment burden |  |  |
|  | Undesirable consequences clearly outweigh desirable consequences in most settings | Persons with pulmonary TB are anticipated to be most infectious prior to treatment initiation, with rapid reduction in infectiousness with effective treatment irrespective of bacteriologic studies during treatment. The specific duration of treatment at which most PWTB are non-infectious is uncertain. Available data suggests steady declines in infectiousness with longer durations of treatment. The rate at which infectiousness declines with effective treatment is uncertain.  Whether infectiousness persists for longer durations in individuals with higher pre-treatment bacterial burden compared to lower bacterial burden is uncertain. The anticipated benefits of RIR with longer durations greater than five days of treatment are uncertain. The anticipated undesirable consequences of RIR are expected to increase with longer duration.  The balance between desirable and undesirable consequences was judged to be closely balanced after five days of effective treatment in persons with high pre-treatment bacterial burden, with undesirable consequences probably outweighing desirable consequences in most settings. Given variability, the balance of effects may differ in individual circumstances (see footnote 3, Table 3) |
|  | Undesirable consequences probably outweigh desirable consequences in most settings |  |
|  | **The balance between desirable and undesirable consequences is closely balanced or uncertain** |  |
|  | Desirable consequences probably outweigh undesirable consequences in most settings |  |
|  | Desirable consequences clearly outweigh undesirable consequences in most settings |  |
|  |  |  |
| Treatment>= 5 days, low pre-treatment burden |  |  |
|  | Undesirable consequences clearly outweigh desirable consequences in most settings | Persons with pulmonary TB are anticipated to be most infectious prior to treatment initiation, with rapid reduction in infectiousness with effective treatment irrespective of bacteriologic studies during treatment. The specific duration of treatment at which most PWTB are non-infectious is uncertain. Available data suggests steady declines in infectiousness with longer durations of treatment. The rate at which infectiousness declines with effective treatment is uncertain. The anticipated benefits of RIR with longer durations greater than five days of treatment are uncertain, particularly in individuals with low pre-treatment bacterial burden. The anticipated undesirable consequences of RIR are expected to increase with longer duration.  The undesirable consequences probably outweigh desirable consequences in most settings after five days of effective treatment. Given variability, the balance of effects may differ in individual circumstances (see footnote 3, Table 3) |
|  | **Undesirable consequences probably outweigh desirable consequences in most settings** |  |
|  | The balance between desirable and undesirable consequences is closely balanced or uncertain |  |
|  | Desirable consequences probably outweigh undesirable consequences in most settings |  |
|  | Desirable consequences clearly outweigh undesirable consequences in most settings |  |
|  |  |  |
| Treatment>= 14 days, low or high pre-treatment burden |  |  |
|  | Undesirable consequences clearly outweigh desirable consequences in most settings | Persons with pulmonary TB are anticipated to be most infectious prior to treatment initiation, with rapid reduction in infectiousness with effective treatment irrespective of bacteriologic studies during treatment. The specific duration of treatment at which most PWTB are non-infectious is uncertain. Available data suggests steady declines in infectiousness with ongoing treatment. The anticipated undesirable consequences of RIR are expected to increase with longer duration.  The undesirable consequences probably outweigh desirable consequences in most settings with greater than 14 days of effective treatment for both individuals with low and high pre-treatment bacterial burden. |
|  | **Undesirable consequences probably outweigh desirable consequences in most settings** |  |
|  | The balance between desirable and undesirable consequences is closely balanced or uncertain |  |
|  | Desirable consequences probably outweigh undesirable consequences in most settings |  |
|  | Desirable consequences clearly outweigh undesirable consequences in most settings |  |
|  |  |  |
|  |  |  |

1.	Macq J, Solis A, Martinez G, Martiny P. Tackling tuberculosis patients' internalized social stigma through patient centred care: an intervention study in rural Nicaragua. BMC Public Health **2008**; 8: 154.

2.	Van Rie A, Sengupta S, Pungrassami P, et al. Measuring stigma associated with tuberculosis and HIV/AIDS in southern Thailand: exploratory and confirmatory factor analyses of two new scales. Trop Med Int Health **2008**; 13(1): 21-30.

3.	U.S. Department of Housing and Urban Development. Measuring Housing Insecurity: Index Development Using American Housing Survey Data. Available at: <https://www.huduser.gov/portal/publications/Measuring-Housing-Insecurity-Index-Development-Using-AHS-Data.html>. Accessed November 2023.

4.	Blumberg SJ, Bialostosky K, Hamilton WL, Briefel RR. The effectiveness of a short form of the Household Food Security Scale. Am J Public Health **1999**; 89(8): 1231-4.

5.	USDA. U.S. Household Food Security Survey Module: Six-Item Short Form  Economic Research Service. Available at: <https://www.ers.usda.gov/media/8282/short2012.pdf>. Accessed November 2023.

6.	Vander Elst T, De Witte H, De Cuyper N. The Job Insecurity Scale: A psychometric evaluation across five European countries. European Journal of Work and Organizational Psychology **2014**; 23(3): 364-80.

7.	Center PR. The Politics of Financial Insecurity: A Democratic Tilt, Undercut by Low Participation. Available at: <https://www.pewresearch.org/politics/2015/01/08/the-politics-of-financial-insecurity-a-democratic-tilt-undercut-by-low-participation/>. Accessed January 2024.

8.	Kroenke K, Spitzer RL, Williams JB. The PHQ-9: validity of a brief depression severity measure. J Gen Intern Med **2001**; 16(9): 606-13.

9.	Kroenke K, Spitzer RL. The PHQ-9: A New Depression Diagnostic and Severity Measure. Psychiatric Annals **2013**; 9(32): 509-15.

10.	Beck AT, Ward CH, Mendelson M, Mock J, Erbaugh J. An inventory for measuring depression. Arch Gen Psychiatry **1961**; 4: 561-71.

11.	The Links Between Tuberculosis and Mental Health: Evidence and Best Practice Incorporating Guidance to USAID. Available at: <https://www.usaid.gov/sites/default/files/2022-05/TB_and_Mental_Health_USAID_REPORT_FINALTRACKED_EDITS_AG_508c_1.pdf>. Accessed November 2023.

12.	American Psychiatric Association. DSM-5-TR Online Assessment Measures. Available at: <https://www.psychiatry.org/psychiatrists/practice/dsm/educational-resources/assessment-measures>. Accessed January 2024.

13.	Guyatt GH, Oxman AD, Vist GE, et al. GRADE: an emerging consensus on rating quality of evidence and strength of recommendations. Bmj **2008**; 336(7650): 924-6.

14.	Michael JB, Tracy AW, Lori P, et al. Putting Evidence Into Practice: An Update on the US Preventive Services Task Force Methods for Developing Recommendations for Preventive Services. The Annals of Family Medicine **2023**; 21(2): 165.

15.	Advisory Committee on Immunization Practice. ACIP Evidence to Recommendation User’s Guide. Available at: <https://www.cdc.gov/vaccines/acip/recs/grade/downloads/acip-evidence-rec-frame-user-guide.pdf>. Accessed January 2024.

16.	Guyatt GH, Schünemann HJ, Djulbegovic B, Akl EA. Guideline panels should not GRADE good practice statements. Journal of Clinical Epidemiology **2015**; 68(5): 597-600.

17.	Hilton Boon M, Thomson H, Shaw B, et al. Challenges in applying the GRADE approach in public health guidelines and systematic reviews: a concept article from the GRADE Public Health Group. J Clin Epidemiol **2021**; 135: 42-53.

18.	Atkins D, Best D, Briss PA, et al. Grading quality of evidence and strength of recommendations. Bmj **2004**; 328(7454): 1490.

19.	Canadian Tuberculosis Standards. Vol. 6:sup1, 1-7: Canadian Journal of Respiratory, Critical Care, and Sleep Medicine, **2022**.

20.	Balshem H, Helfand M, Schünemann HJ, et al. GRADE guidelines: 3. Rating the quality of evidence. Journal of Clinical Epidemiology **2011**; 64(4): 401-6.

21.	Reeve BWP, McFall SM, Song R, Warren R, Steingart KR, Theron G. Commercial products to preserve specimens for tuberculosis diagnosis: a systematic review. Int J Tuberc Lung Dis **2018**; 22(7): 741-53.

22.	Centers for Disease Control and Prevention. Reported Tuberculosis in the United States, 2021. Available at: <https://www.cdc.gov/tb/statistics/reports/2021/default.htm>. Accessed March 23, 2024.
